# Supramolecular template-directed synthesis of triazole oligomers†

**DOI:** 10.1039/d2sc04155c

**Published:** 2022-11-01

**Authors:** Francesco Fasano, Peter Bolgar, Giulia Iadevaia, Christopher A. Hunter

**Affiliations:** Yusuf Hamied Department of Chemistry, University of Cambridge Lensfield Road Cambridge CB2 1EW UK herchelsmith.orgchem@ch.cam.ac.uk

## Abstract

Sandwich complexes formed by two zinc porphyrins and a diamine ligand (DABCO) have been used as a supramolecular template to direct the synthesis of triazole oligomers. Monomer units equipped with two polymerizable functional groups, an alkyne and an azide, were attached to the template *via* ester bonds between a phenol unit on the monomer and benzoic acid units on the porphyrin. Self-assembly of the zinc porphyrins by addition of DABCO led to a supramolecular complex containing four of the monomer units, two on each porphyrin. CuAAC oligomerisation was carried out in the presence of a chain capping agent to prevent intermolecular reactions between the templated products, which carry reactive chain ends. The templated-directed oligomerisation resulted in selective formation of a duplex, which contains two identical chains of triazole oligomers connecting the porphyrin linkers. The effective molarity for the intramolecular CuAAC reactions on the template is 3–9 mM, and because the triazole backbone has a direction, the product duplex was obtained as a 4 : 1 mixture of the parallel and antiparallel isomers. Hydrolysis of the ester bonds connecting the oligomers to the template gave a single product, the phenol 2-mer, in excellent yield. The introduction of a supramolecular element into the template considerably broadens the scope of the covalent template-directed oligomerisation methodology that we previously developed for the replication of sequence information in synthetic oligomers.

## Abstract 2

A supramolecular metalloporphyrin assembly was used as a disposable template for controlling the oligomerisation of covalently attached monomer building blocks to give a linear oligomeric product that is not accessible *via* untemplated reactions.

## Graphical abstract

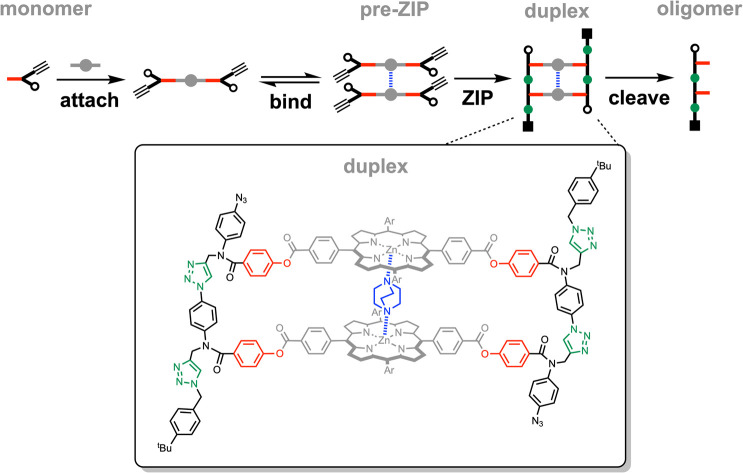

## Introduction

Template-directed synthesis is a powerful strategy to control the formation of complex molecular architectures.^1–5^ In the template synthesis of macrocyclic oligomers significant progress has been made since the pioneering work of Pederson.^6–13^ Templating the synthesis of linear oligomers involves an additional level of challenge, because the product contains reactive terminal groups that are not present in the corresponding macrocycles. Further oligomerisation of linear products must therefore be blocked by controlling the reaction time or concentration, or by using end-capping reagents.^14–16^ Over the past few decades, many efforts have been made to tackle the challenges in the synthesis of non-natural linear oligomers.^2,17–19^

**Fig. 1:**
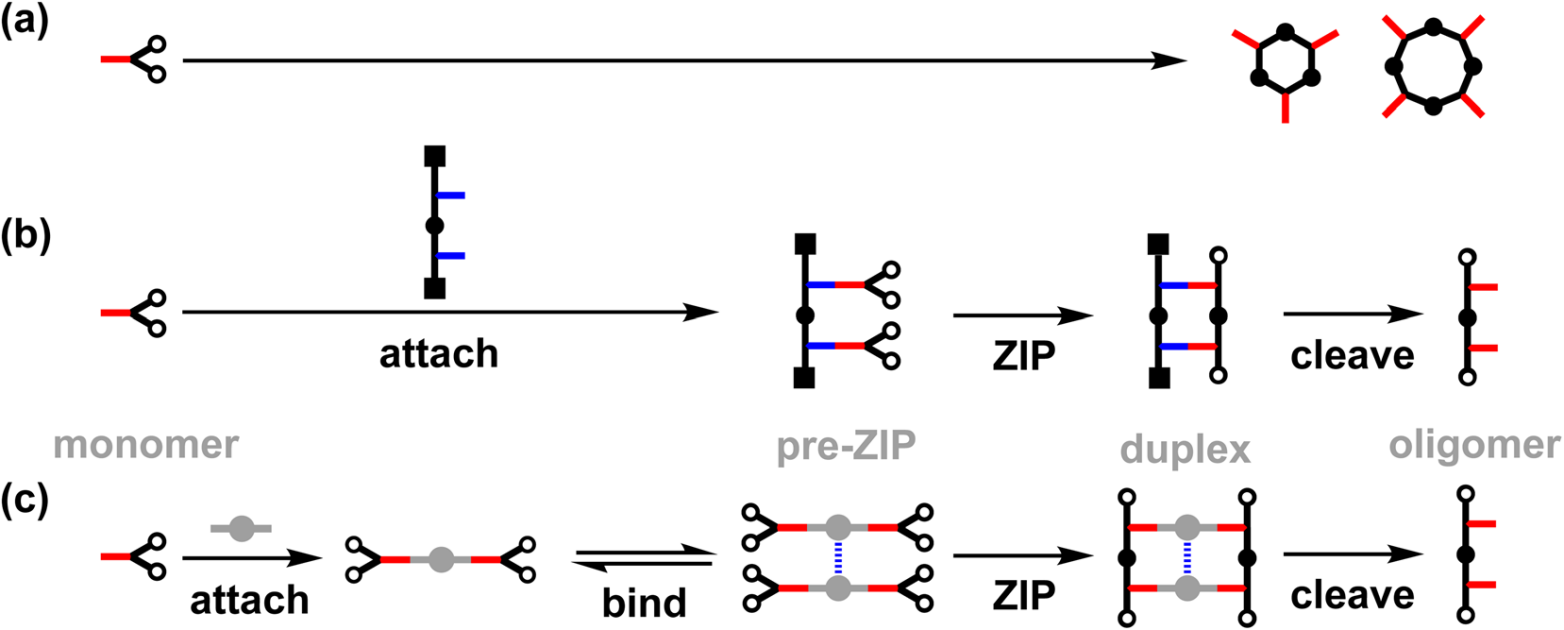
(a) Untemplated oligomerization of the monomer (red) gives a mixture of cyclic oligomers. (b) Covalent template-directed synthesis. In the *attach* step, monomers are covalently linked to complementary sites on the template (blue). In the *ZIP* step, intramolecular reactions between the monomers leads to oligomerization. In the *cleave* step, the new oligomer is released from the template. (c) Supramolecular template-directed synthesis. In the *attach* step, monomers are covalently attached to a linker (grey). In the *bind* step, linkers are connected *via* non-covalent interactions. In the *ZIP* step, intramolecular reactions between the monomers leads to oligomerization. In the *cleave* step, the new oligomer is released from the template.

We have been working on an approach to template-directed oligomer synthesis based on covalent base-pairing (Fig. 1).^20,21^ The monomer building blocks (red) are attached to complementary sites (blue) on a template strand using kinetically stable covalent bonds to give a pre-ZIP intermediate. In the ZIP step, an oligomerisation reaction on the template gives the duplex. Finally, cleavage of the base-pairs releases the original template and the complementary copy. We have shown that this strategy can be successfully implemented in high yield by using phenol–benzoic acid esters for the base-pairing chemistry and copper-catalysed azide–alkyne cycloaddition (CuAAC) for the backbone ZIP reaction.^22–24^ In the absence of a template, macrocyclic 3-mers and 4-mers are the major products (Fig. 1a), but a 2-mer template can be used to direct exclusive formation of the linear 2-mer (Fig. 1b). This approach to oligomer synthesis is attractive because the sequence of the copy strand can be defined by the sequence of the template. However, separate synthesis of an oligomeric template is always required. Fig. 1c shows an alternative strategy for assembling monomers on a supramolecular template for oligomerisation.

In the first step of the supramolecular strategy shown in Fig. 1c, two monomers (red) are covalently attached to a disposable linker (grey). Dynamic non-covalent binding interactions between the linker units are then used to self-assemble an oligomeric pre-ZIP intermediate. The ZIP step forms both backbones of the duplex simultaneously. Finally, the cleave step gives the oligomeric product, where the length is defined by the number of units in the self-assembled pre-ZIP template. Here we demonstrate the viability of this strategy with the synthesis of 2-mers on a zinc porphyrin–DABCO (1,4-diazabicyclo[2.2.2]octane) template. Complexes of this type have been used previously for templated synthesis of cages,^25^ and metalloporphyrin coordination chemistry is compatible with metal-catalysed coupling reactions.^26–32^ Porphyrin–ligand complexes allow considerable flexibility, because different divalent ligands can be used to match the geometry of different duplex backbones, and different metal–ligand combinations can be used to tune the stability and length of the pre-ZIP intermediate.^33–38^

## Results

### Synthesis

**Scheme 1:**
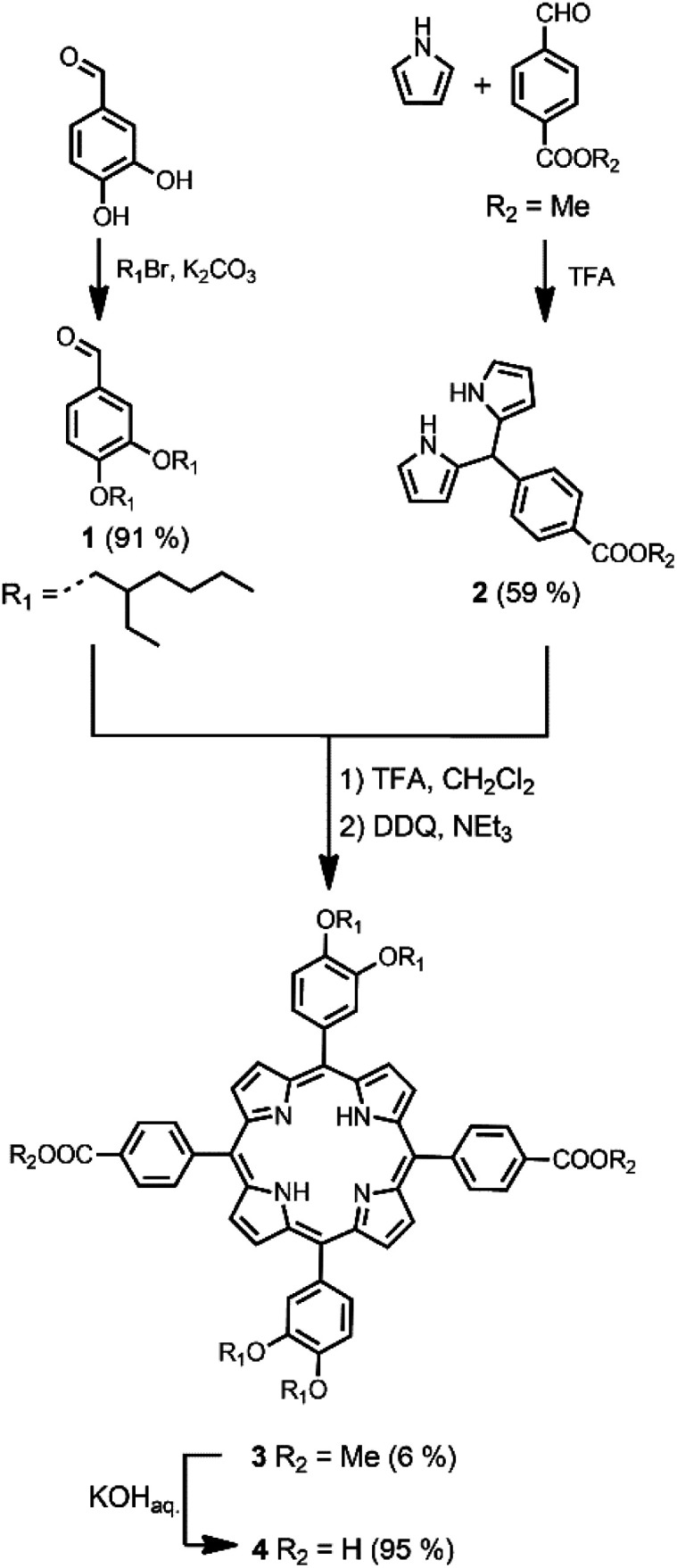
Synthesis of free base porphyrin 4.

The synthetic route to the disposable porphyrin linker is shown in Scheme 1. 3,4-Dihydroxybenzaldehyde was alkylated with racemic 2-ethylhexyl bromide to give 1. Reaction of pyrrole with 4-(methoxycarbonyl)benzaldehyde in TFA gave dipyrromethane 2. Compounds 1 and 2 were condensed using TFA catalysis to give porphyrin 3, and hydrolysis of the esters gave the porphyrin dicarboxylic acid 4. The use of four 2-ethylhexyl substituents gives rise to a diastereomeric mixture of compounds, ensuring good solubility in non-polar solvents.

**Scheme 2:**
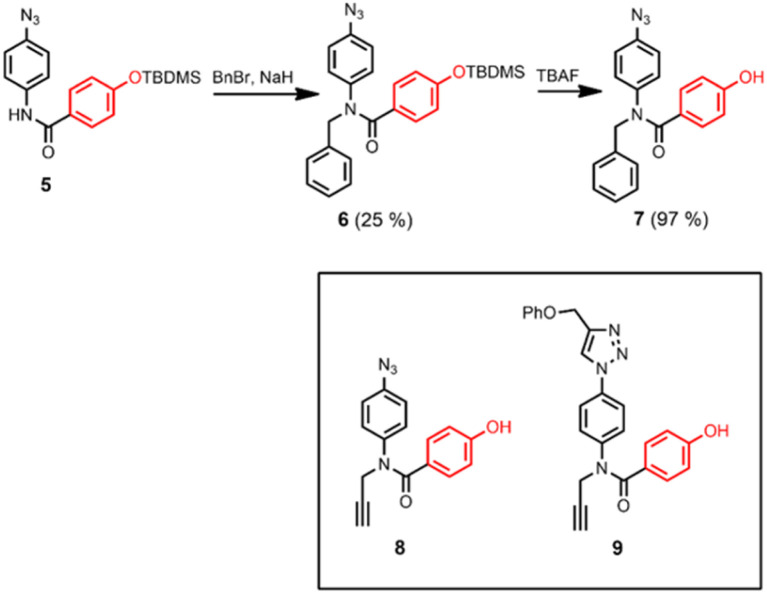
Synthesis of 7, and structures of 8 and 9.

**Scheme 3:**
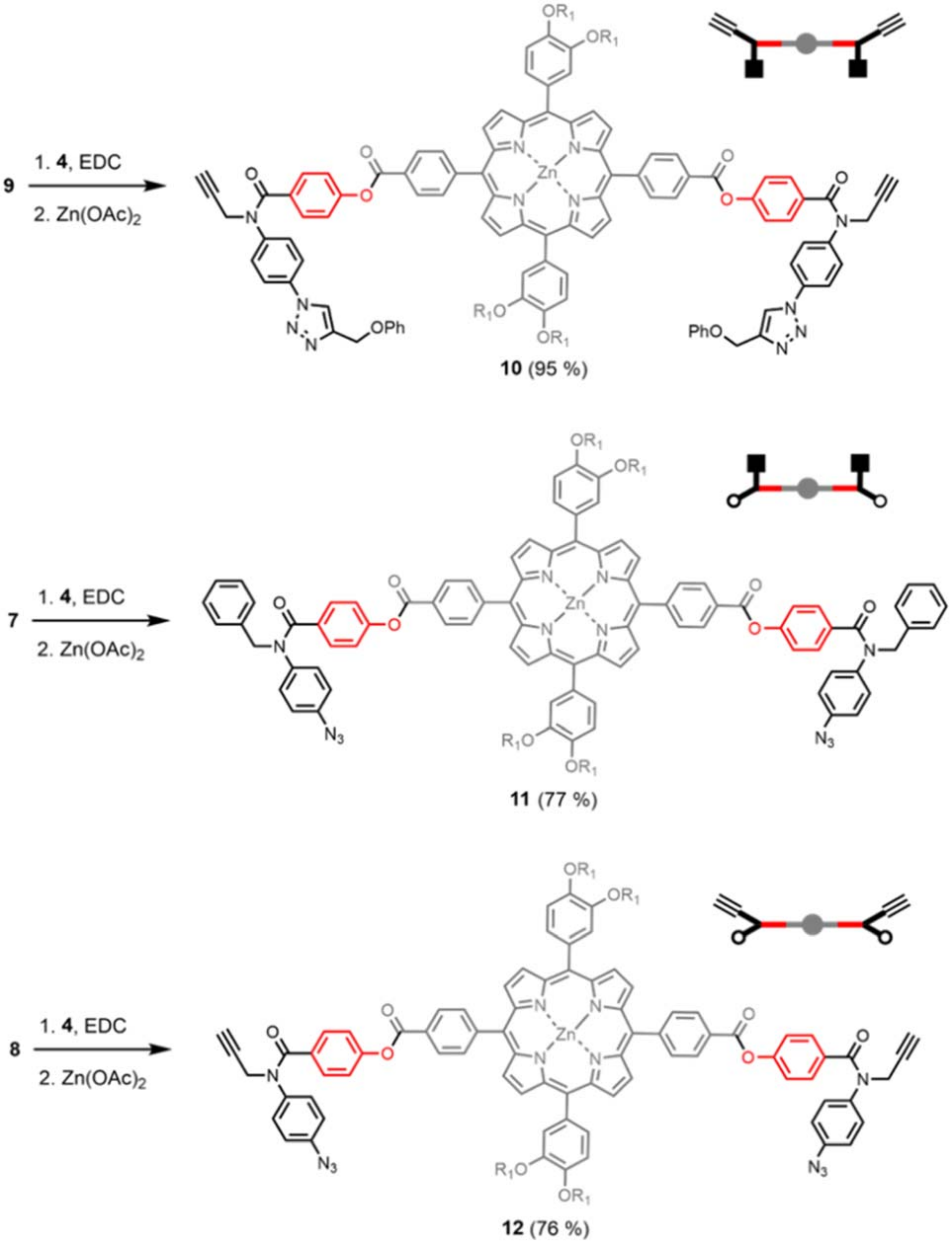
Synthesis of zinc porphyrins 10, 11 and 12.

Scheme 2 shows three different phenol monomer building blocks: 7 is equipped with only an azide, 8 has both an azide and an alkyne, and 9 has only an alkyne. The synthesis of 8 and 9 have been described previously.^20^ Phenol 7 was prepared by reaction of 5 with benzyl bromide in the presence of sodium hydride, followed by TBAF deprotection (Scheme 2). The three phenol monomers were separately loaded onto porphyrin linker 4 using EDC coupling reactions, and the resulting porphyrins were then metalled with zinc acetate (Scheme 3). In zinc porphyrin 12, the linker is loaded with phenol monomers equipped with both an alkyne and azide, which would lead to polymers in the absence of a template under CuAAC conditions. In zinc porphyrin 10, the phenols are equipped with alkynes only, and in zinc porphyrin 11, the phenols are equipped with azides only. Thus the reaction between 10 and 11 can be used to investigation template effect of bidentate ligands on the CuAAC reaction without the complication of competing polymerisation processes.

### Templated reaction of 10 with 11

**Fig. 2:**
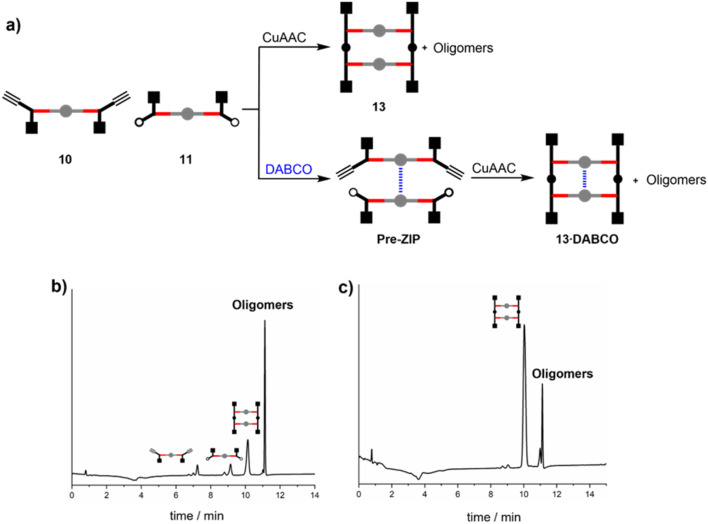
(a) Reaction pathways in the CuAAC reaction between 10 and 11 in the absence or presence of DABCO. HPLC chromatograms of the product mixture obtained from reaction of 10 (15 μM), 11 (15 μM) and CuTBTA (0.02 mM) in CH_2_Cl_2_ at room temperature for 16 h (b) in the absence of DABCO, and (c) in the presence of DABCO (15 μM). The sharp peak at 11 minutes corresponds to the point at which the eluant switched to 100% THF and washed all remaining species off the column.

**Fig. 3:**
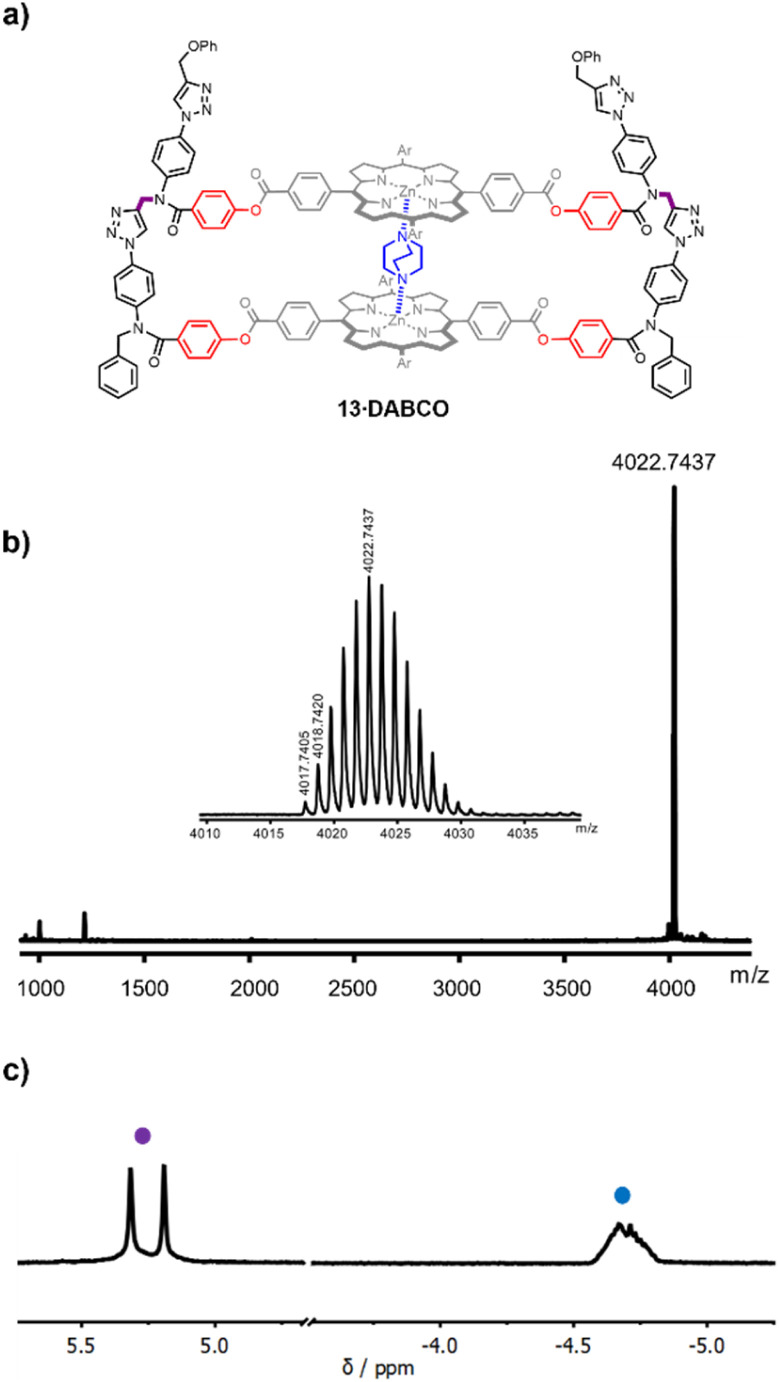
(a) Chemical structure of the DABCO complex of duplex 13. (b) MALDI-HRMS of 13 (calculated mass for [M]^+^ = 4017.7608). (c) Partial 500 MHz ^1^H NMR spectrum of the DABCO complex of 13 in CDCl_3_ at room temperature. The signals due to the amide NCH_2_ groups (purple), and the upfield shifted signal due to bound DABCO (blue) are indicated.

Zinc porphyrins 10 and 11 were reacted under CuAAC conditions in the presence and absence of DABCO in dichloromethane solution (Fig. 2a). The HPLC trace of the crude reaction mixture in the absence of DABCO (Fig. 2b) shows that the ZIP reaction did not proceed to complete conversion. After 16 hours of reaction, the starting materials 10 and 11 were still present. The major product was a mixture of higher oligomers, and relatively small amounts of the macrocyclic duplex 13 were observed. Addition of 0.5 equivalents of DABCO relative to the total amount of zinc porphyrin should lead to self-assembly of a mixture of 2 : 1 sandwich complexes, including the pre-ZIP complex illustrated in Fig. 2a. When the CuAAC reaction between zinc porphyrins 10 and 11 was carried in the presence 0.5 equivalents of DABCO, the starting materials were completely consumed, and the major product was duplex 13 with small amounts of higher oligomers (Fig. 2c). In other words, DABCO acts as a positive template, promoting the formation of duplex at the expense of untemplated oligomerisation processes. This result shows that the spacing of the porphyrin linkers in the 2 : 1 DABCO sandwich complex is compatible with the length of the triazole backbone used in these compounds (Fig. 3a).

The DABCO complex of duplex 13 was isolated in 79% yield from the crude reaction mixture by recycling gel permeation chromatography (see ESI†). The HRMS-MALDI spectrum shown in Fig. 3b confirmed the presence of 13, but the ionisation conditions are too harsh for the intact DABCO complex to be observed. The presence of DABCO bound to 13 was confirmed by the ^1^H-NMR spectrum of the product obtained from the CuAAC reaction (Fig. 3c). The signal due to the DABCO protons appears as an upfield shifted multiplet between −5.0 and −4.5 ppm (blue). This chemical shift is characteristic of a zinc porphyrin·DABCO·zinc porphyrin sandwich complex, where the DABCO protons experience the combined ring current effects of both porphyrin π-systems. The multiplicity of the signal suggests that there are multiple conformations of the duplex, which are in slow exchange on the ^1^H NMR timescale, probably due to slow rotation around the four amide bonds in the constrained macrocyclic structure. However, these conformational isomers do not affect the signals due to the amide NCH_2_ groups (purple), which appear as two singlets between 5.0 and 5.5 ppm (Fig. 3c).

**Fig. 4:**
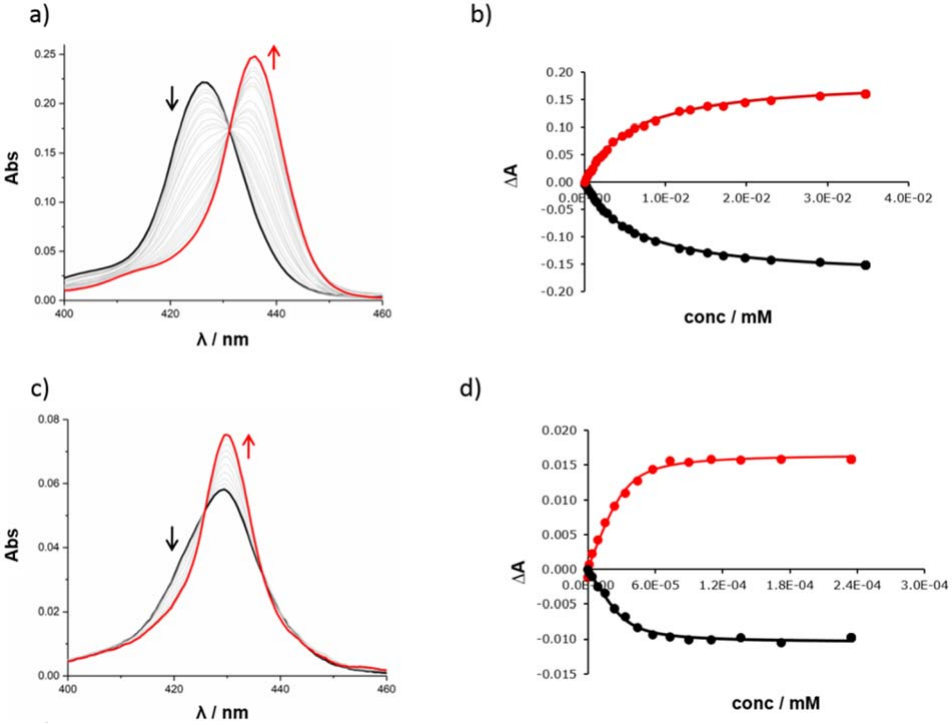
UV-visible titration experiments. (a) UV-visible absorption spectrum for titration of DABCO into 10 (0.38 μM) in chloroform at 298 K. (b) Best fit of the titration data for 10 to a 1 : 1 binding isotherm: absorption measured at 425 nm (black) and 436 nm (red). (c) UV-visible absorption spectrum for titration of DABCO into 13 (0.029 μM) in chloroform at 298 K. (d) Best fit of the titration data for 13 to a 1 : 1 binding isotherm: absorption measured at 423 nm (black) and 429 nm (red).

The high yield of duplex in the templated reaction and the fact that the DABCO complex survives chromatography suggest that the 13·DABCO complex is exceptionally stable. It was possible to remove the DABCO with formic acid to obtain duplex 13. The association constants for the interaction of DABCO with 13 and with the corresponding zinc porphyrin monomer 10 were measured using UV-visible absorption spectroscopy in chloroform (Fig. 4). On addition of DABCO to 10, the Soret band of the zinc porphyrin at 426 nm was replaced by an absorption at 436 nm due to the 1 : 1 DABCO complex with a clear isosbestic point, indicating a simple two-state equilibrium (Fig. 4a). The data fit well to a 1 : 1 binding isotherm (Fig. 4b) giving an association constant of 1.8 × 10^5^ M^−1^, which is typical of a 1 : 1 zinc porphyrin·DABCO complex.^39^ On addition of DABCO to 13, a new Soret band appeared at 430 nm, and the exciton shift of 6 nm relative to the DABCO·10 complex is characteristic of formation of a zinc porphyrin·DABCO·zinc porphyrin sandwich complex (Fig. 4c). A clear isosbestic point was observed, and the data fit well to a 1 : 1 binding isotherm (Fig. 4d). The resulting association constant is three orders of magnitude larger than the value measured for 10 (*K* = 2.1 × 10^8^ M^−1^), which confirms cooperative formation of two zinc-nitrogen coordination bonds in the complex.^39^ These experiments are consistent with the templating results and suggest that DABCO is a good fit for triazole backbone.

### Templated oligomerisation of 12

**Fig. 5:**
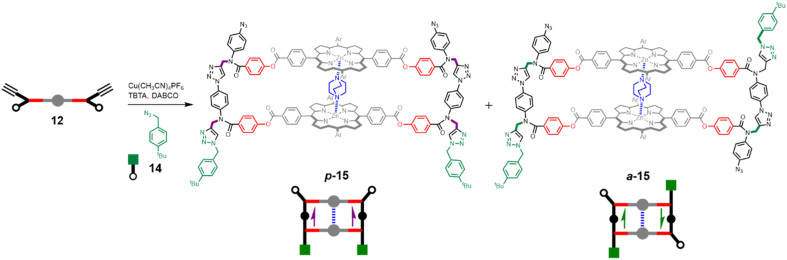
DABCO templated synthesis of duplexes p-15 and a-15. The relative orientations of backbones are indicated with purple arrows for parallel and green arrows for antiparallel.

Having established that DABCO is a good template for the CuAAC reaction, we next investigated the ZIP reaction using zinc porphyrin 12, where the monomer units are equipped with both an alkyne and an azide, so a large number of different products are possible. Fig. 5 illustrates templated formation of the target duplex. There are two important differences compared with the CuAAC reaction of 10 with 11. After the templated ZIP reaction takes place, each chain of the duplex has a terminal alkyne and a terminal azide, so further intermolecular reactions are possible, which would lead to uncontrolled polymerisation. We have shown previously that by working under high dilution conditions (15 μM) in the presence of a large excess of 4-*tert*-butylbenzyl azide 14 it is possible to selectively cap the terminal alkyne of a template-directed oligomerisation reaction, and the same strategy was used here.^14^Fig. 5 shows that two different product duplexes are possible in this case. The triazole backbone has a direction, so there are isomeric products, which differ in the relative orientation of the two backbones, *i.e.* the duplex can be parallel or antiparallel. Duplex 13, which was formed by reacting 10 with 11, has the same backbone orientation as the parallel isomer, p-15.

**Fig. 6:**
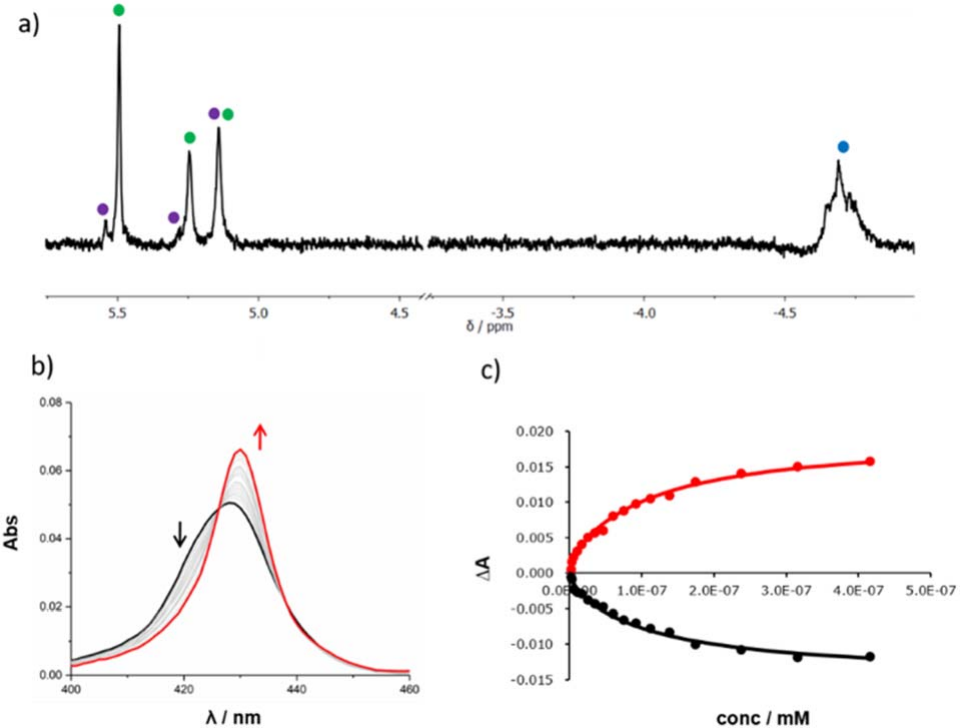
(a) Partial 500 MHz ^1^H NMR spectrum of the DABCO complex of 15 in CDCl_3_ at room temperature. The signals due to the NCH_2_ groups (green and purple), and the upfield shifted signal due to bound DABCO (blue) are indicated. (b) UV-visible absorption spectrum for titration of DABCO into 15 (0.027 μM) in chloroform at 298 K. (c) Best fit of the titration data to a binding isotherm that assumes there are two different duplexes a-15 and p-15, which are present in different amounts and each form a 1 : 1 complex with DABCO with different association constants: absorption measured at 422 nm (black) and 430 nm (red).

Templated oligomerisation of 12 in the presence of 0.5 equivalents of DABCO and 0.4 mM 14 gave the DABCO complexes of 15 in 48% yield after isolation by size exclusion chromatography (Fig. 5). The ^1^H-NMR spectrum of the product in Fig. 6a shows a multiplet due to the DABCO protons between −5.0 and −4.5 ppm (blue), which is indicative of a zinc porphyrin·DABCO·zinc porphyrin sandwich complex and is very similar to the spectrum obtained for the DABCO complex of duplex 13 (*cf.*Fig. 3c). The NCH_2_ region of the ^1^H NMR spectrum shows three major signals (green) as well as a set of minor signals (purple) between 5 and 6 ppm. This observation is consistent with the formation of two isomeric duplexes, a-15 and p-15, in different amounts, and integration of the signals gives a product distribution of 80 : 20.^14^

DABCO was removed from the products by washing with aqueous formic acid, but the resulting mixture of the two isomeric duplexes a-15 and p-15 could not be separated by chromatography. A UV-visible titration was therefore carried out by adding DABCO to this mixture. A new Soret band was observed at 430 nm, characteristic of a zinc porphyrin·DABCO·zinc porphyrin sandwich complex (Fig. 6b). We assume that the association constant for DABCO binding to the parallel duplex p-15 is the same as that for binding to duplex 13, because both have parallel backbones and the only differences in structure are the peripheral groups on the chain ends. The titration data for 15 could therefore be fit to an isotherm that allowed for formation of two different 1 : 1 complexes (Fig. 6c). The association constant for p-15 was fixed at the value measured for 13 (*K*_p_ = 2.1 × 10^8^ M^−1^), and the association constant for a-15 was optimised giving a value of *K*_a_ = 8.6 × 10^6^ M^−1^. In addition, the relative proportion of the two isomers was treated as a variable, and optimisation of this parameter gave a product distribution of 80% a-15 and 20% p-15, which is in good agreement with the integration of the ^1^H NMR signals.

It is worth noting that the duplex obtained in highest yield from the templating process has a slightly lower affinity for DABCO. There must be a difference between the geometry of the triazole backbone in the product duplex compared with the geometry of the transition state of the templated CuAAC reaction, which leads to this difference in behaviour.

### Effective molarity of the ZIP reaction

**Fig. 7:**
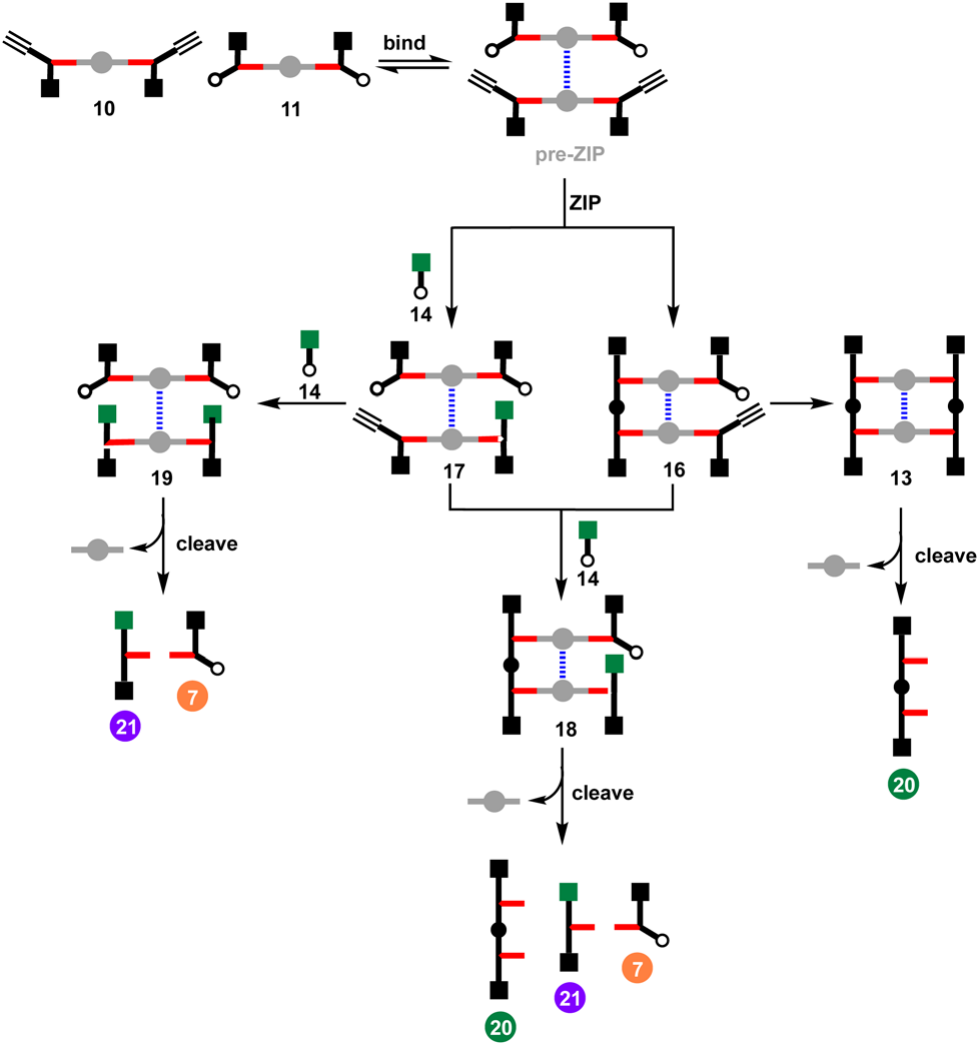
Cartoon representation of reaction pathways in the CuAAC ZIP reaction of 10 and 11 in the presence of DABCO and capping agent 14, followed by ester hydrolysis to cleave the products from the porphyrin linker.

The effective molarity (EM) for the intramolecular ZIP reaction that takes place in the DABCO complex can be determined by carrying out a competition reaction with an externally added reagent. As we have shown previously, increasing the concentration of the capping azide 14 provides a convenient method for measuring the value of EM for intramolecular CuAAC reactions.^14^ The approach is illustrated in Fig. 7 for the reaction between 10 and 11 in the presence of DABCO. There are two intramolecular reactions in the ZIP step, and 14 could intercept either or both reactions. In the first CuAAC reaction, one of the two alkynes in the pre-ZIP intermediate will either react in an intramolecular fashion to form 16 or react with the capping azide to form 17. The second alkyne in 16 can again react in an intramolecular fashion give duplex 13 or in an intermolecular fashion to give 18. The second alkyne in 17 can either react intramolecularly to give 18 or intermolecularly with the capping azide to give 19. Analysis of the product distribution was simplified by cleaving the esters to release the phenol groups from the porphyrin linker giving three different products, the phenol 2-mer 20, the starting azide monomer 7, and the capped alkyne monomer 21.

**Fig. 8:**
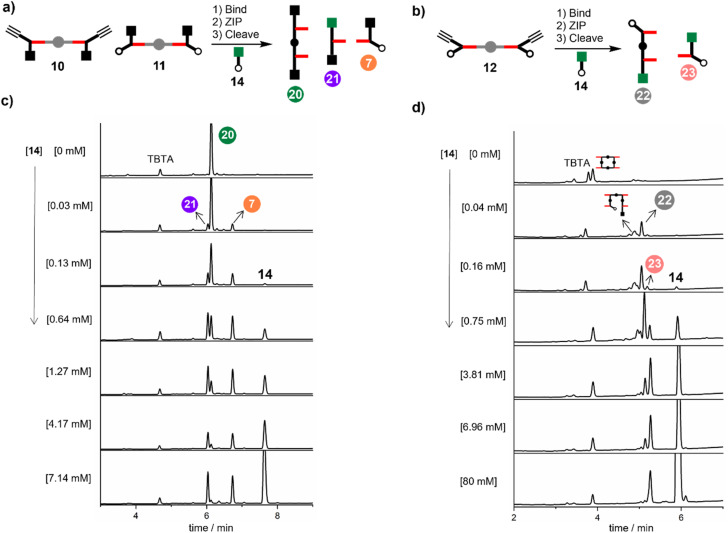
(a) Cartoon representation of products obtained from the CuAAC ZIP reaction of 10 and 11 in the presence of DABCO and capping agent 14, followed by ester hydrolysis to cleave the porphyrin linker. (b) Cartoon representation of products obtained from the CuAAC ZIP reaction of 12 in the presence of DABCO and capping agent 14, followed by ester hydrolysis to cleave the porphyrin linker. (c) HPLC chromatograms showing the effect of capping agent 14 on the product distribution obtained from reaction of 10 (15 μM), 11 (15 μM), DABCO (15 μM) and CuTBTA (0.02 mM) in CH_2_Cl_2_ at room temperature for 16 h followed by hydrolysis with LiOH (1 M). (d) HPLC chromatograms showing the effect of capping agent 14 on the product distribution obtained from reaction of 12 (15 μM) DABCO (7.5 μM) and CuTBTA (0.02 mM) in CH_2_Cl_2_ at room temperature for 16 h followed by hydrolysis with LiOH (1 M).

Fig. 8a shows the result of carrying out the CuAAC reaction followed by ester hydrolysis for a 1 : 1 : 1 mixture of 10, 11 and DABCO in the presence of increasing amounts of 14. In the absence of 14, only the phenol 2-mer 20 was observed. When millimolar concentrations of capping azide were introduced, a mixture of all three products was observed. At higher concentrations of 14 (7 mM), the intramolecular reaction was completely suppressed, and the only products observed were 21 and 7. The same experiment was carried out for a 2 : 1 mixture of 12 and DABCO in the presence of increasing amounts of capping azide 14 (Fig. 8b). In the absence of 14, only oligomeric products were obtained, because after the intramolecular ZIP reactions take place, the terminal alkyne and azide groups on the duplex continue to react in an intermolecular fashion. Small amounts of capping agent (<1 mM) suppress these intramolecular processes, so that the phenol 2-mer 22 was the only major product obtained after hydrolysis. At higher concentrations of capping agent, intermolecular reactions with 14 start to compete with the intramolecular ZIP process, leading to formation of the capped monomer 23.

**Fig. 9:**
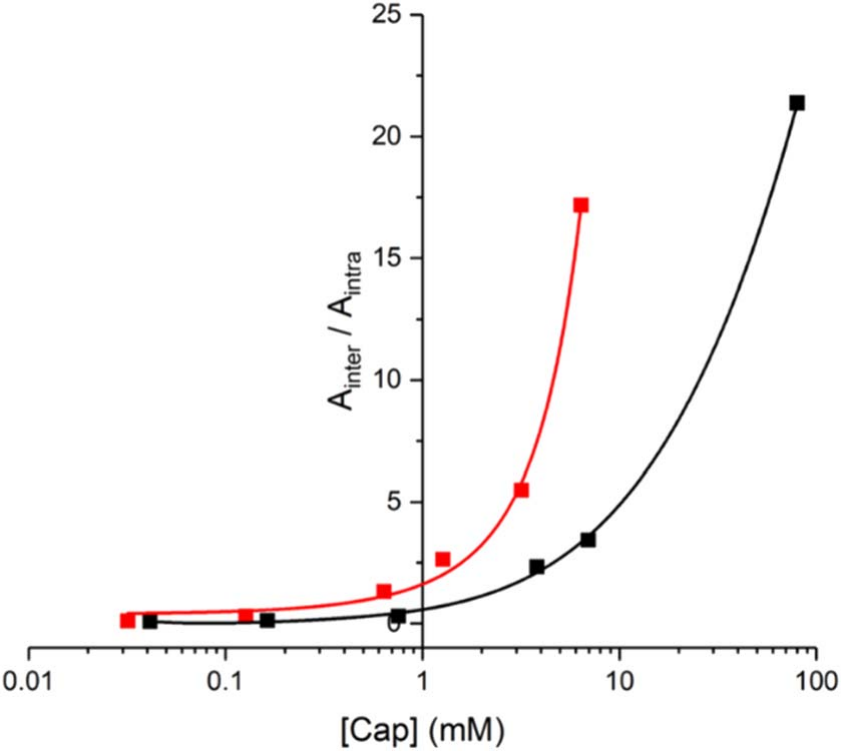
Product distribution for the templated reaction of 10 with 11 (red), and for the templated oligomerisation of 12 (black) in the presence of different concentrations of 14 ([cap]). The data are plotted as the ratio of the area of the HPLC peaks assigned to the product of the intermolecular reaction with the capping agent (*A*_inter_ for 21 and 23) compared with the area of the HPLC peaks assigned to the product of the intramolecular ZIP reaction (*A*_intra_ for 20 and 22). Reactions were carried out using 10 (15 μM) and 11 (15 μM), or 12 (15 μM), with CuTBTA (0.02 mM) in CH_2_Cl_2_ at room temperature for 16 h, followed by hydrolysis with LiOH (1 M). The lines represent the best fit to eqn (1).

In principle, the EM can be determined from the concentration of capping agent at which the yields of products due to intermolecular and intramolecular processes are equal (*i.e.* monomer and 2-mer). However, there is a 5-fold difference in reactivity between the aliphatic capping azide and the aromatic azides on the phenol monomers, which must also be taken into account.^14^ The relationship between the product distribution and the concentration of capping agent is shown in Fig. 9. Since the intermolecular process results in two phenol monomers, the integrated absorbance of the HPLC peak due to monomer can be directly compared with the peak due to the 2-mer product to obtain a direct measure of the relative yields of intermolecular *versus* intramolecular processes (eqn (1)).

1 *A*_inter_/*A*_intra_ = 5[cap]/EM

The value of EM for the templated reaction between 10 and 11 is 3 mM, whereas the EM for the templated oligomerisation of 12 is 9 mM. Since 10 and 11 can only form the parallel duplex 13, this result implies that the higher EM observed for 12 is due to preferential formation of the antiparallel duplex a-15. These EM values represent composite values of the two different CuAAC reactions required to assemble the duplex. The reaction to connect the first backbone linkage takes place on the 2 : 1 zinc porphyrin·DABCO·zinc porphyrin sandwich complex, and in this case, there is no parallel-antiparallel issue. The difference between parallel and antiparallel only becomes apparent for 12 on closing the second backbone linkage to give the duplex. The values of EM for the first and second reactions are likely to be different but are difficult to separate. Nevertheless, if we assume that 3 mM and 9 mM represent the values of EM for formation of parallel and antiparallel duplexes respectively, we would predict a product distribution for templated oligomerisation of 12 of 75% a-15 and 25% p-15, which is consistent with the UV-visible titration and ^1^H NMR results. These values of EM for the supramolecular zinc porphyrin·DABCO template can be compared with the corresponding values measured previously for formation of parallel and antiparallel duplexes using the same phenol monomer 8 on a covalent template (Fig. 1b). In this case, the values of EM were one to two orders of magnitude higher, and there was significantly higher selectivity for the antiparallel (EM = 200–500 mM) relative to the parallel (EM = 20–30 mM) duplex.^14^

## Conclusions

The approach outlined here shows that supramolecular assemblies can serve as templates to direct the synthesis of covalent oligomers with excellent precision. The supramolecular assembly process is translated into a covalent structure and the use of cleavable linkers between the template and product means that the template can be removed and recycled at the end of the process. The methodology has been demonstrated using 2 : 1 zinc porphyrin·DABCO·zinc porphyrin sandwich complexes as the supramolecular template. Monomer units equipped with two polymerizable functional groups, an alkyne and an azide, were attached to the template *via* ester bonds between a phenol unit on the monomer and benzoic acid units on the porphyrin. Self-assembly of the zinc porphyrins by addition of DABCO lead to a supramolecular complex containing four of the monomer units, two on each porphyrin. CuAAC oligomerisation was carried out in the presence of a chain capping agent to prevent intermolecular reactions between the templated products, which carry reactive chain ends. The templated-directed oligomerisation resulted in selective formation of a duplex, which contains two identical chains of triazole oligomers connecting the porphyrin linkers. The effective molarity for the intramolecular CuAAC reactions on the template is 3–9 mM, and because the triazole backbone has a direction, the product duplex is obtained as a 4 : 1 mixture of the parallel and antiparallel isomers. Hydrolysis of the ester bonds connecting the oligomers to the template gave a single product, the phenol 2-mer, in excellent yield. These experiments show that supramolecular assemblies can be used as disposable templates for controlling covalent reactions to give products that are inaccessible *via* solution phase oligomerisation processes. The example described here used zinc porphyrins, which coordinate a single ligand on one face of the porphyrin. Cobalt porphyrins can coordinate two ligands, one on each face of the porphyrin, which would lead to supramolecular polymers in the presence of bidentate ligands, and we are currently investigating template-directed synthesis of longer oligomers using this approach.^40^ In more general terms, any supramolecular assembly could be used as the template, so that the oligomerisation reaction provides a method for printing supramolecular assemblies as covalent copies.

## Data availability

All supporting data is provided in the ESI.†

## Author contributions

FF, PB and GI carried out the experiments, and all authors contributed to writing the manuscript.

## Conflicts of interest

There are no conflicts to declare.

## Supplementary Material

SC-013-D2SC04155C-s001
