# Supplementary material for: Supramolecular template-directed synthesis of triazole oligomers
Source: Chem Sci. 2022 Nov 1;13(44):13085–93. doi: 10.1039/d2sc04155c (PMC9667925; doi:10.1039/d2sc04155c)
Supplement: SC-013-D2SC04155C-s001 [file SC-013-D2SC04155C-s001.pdf]

Electronic Supplementary Material (ESI) for Chemical Science.
This journal is © The Royal Society of Chemistry 2022

Supramolecular template-directed synthesis of triazole oligomers

Francesco Fasano, Peter Bolgar, Giulia Iadevaia, Christopher A. Hunter*

Department of Chemistry, University of Cambridge, Lensfield Road, Cambridge CB2 1EW, UK

Supporting Information

General experimental details

All reagents and solvents used for the synthesis of the compounds listed below were bought from commercial sources and used without further purification. Dry THF and CH_2Cl_2 were taken from a solvent purification system (Pure Solv™, Innovative Technology, Inc.). Anhydrous DMF was purchased from Sigma-Aldrich. Thin layer chromatography was carried out using silica gel 60F (Merck). Flash chromatography was carried out on an automated system (Combiflash Rf+ or Combiflash Rf Lumen) using prepacked cartridges of silica (25µ PuriFlash® Columns). LCMS analysis was performed on either an analytical Agilent HP1200 coupled to an Agilent/Bruker ion trap or on a Waters Acquity H-class UPLC coupled with a single quadrupole Waters SQD2. NMR spectra were recorded on Bruker 400 MHz DPX400, 400 MHz AVIII400, 500 MHz DCH cryoprobe or 500 MHz TCI Cryoprobe spectrometer and were internally referenced to the residual proton solvent signal. All chemical shifts (δ) are quoted in ppm and coupling constants given in Hz. Abbreviations for signals multiplicity are: s (singlet), bs (broad singlet), d (doublet), t (triplet), q (quadruplet), m (multiplet). FT-IR spectra were measured on a PerkinElmer Spectrum One spectrometer equipped with an ATR cell. Melting points were measured in a Mettler Toledo MP50 Melting Point System. HRMS analysis was carried out on a Waters LCT Premier equipped with a TOF mass analyser and W optics for enhanced resolution, using 50% aqueous acetonitrile with 0.25% formic acid as mobile phase.

LCMS methods

Chromatographic separation was achieved using a CORTECS C18+, 2.7 µm, 4.6×50 mm column (Waters) for methods A and B. Eluents used were H_2O + 0.1% formic acid as solvent A in all the methods and THF + 0.1% formic acid (methods A) or ACN + 0.1% formic acid (methods B) as solvent B.

LCMS method A

Eluent B was linearly increased from 5 to 70% B in 4 minutes, then from 70% to 75% in 6 minutes and finally up 100%. Flow rate was 0.9 ml/min and the column was kept 40 °C.

LCMS method B

Eluent B was linearly increased from 5 to 70% B in 5.25 minutes, then from 65% to 75% in 3 minutes and finally up 100%. Flow rate was 1 ml/min and the column was kept 40 °C.

General procedure for kinetic experiments of CuAAC and hydrolysis reactions

The calculated amount from stock solutions of pre-ZIP intermediate, capping azide (when present) and Cu(I) catalyst were transferred in this order under nitrogen in a double neck flask containing dry THF. Catalyst stock solutions were prepared by mixing equal moles of $Cu(CH_3CN)_4PF_6$ and TBTA (CuTBTA). All stock solutions were freshly prepared in dry THF and used under a stream of nitrogen. Method A was used to follow the formation of ***p*-13** and ***a,p*-15** (ZIP step). Reaction progress was followed by injecting 3 µl of unquenched reaction mixture collected at specific time intervals in the LCMS and observing the change in the peak areas of reagents and products by UV (λ = 420 nm). Peaks identity was assigned based on the corresponding mass spectra, order of appearance and retention time. Method B was used to follow the hydrolysis reaction (cleave step). Reaction progress was followed by injecting 10 µl of unquenched reaction mixture collected at specific time intervals in the LCMS and observing the change in the peak areas of reagents and products by UV (λ = 230 nm). Peaks identity was assigned based on the corresponding mass spectra, order of appearance and retention time.

Synthetic procedures and spectral data

Synthesis of 3,4-bis(2-ethylhexoxy)benzaldehyde [1]

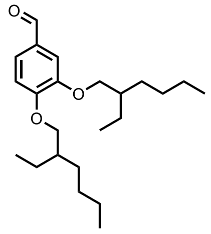

1

To a suspension of 3,4-dihydroxybenzaldehyde (1 g, 7.24 mmol) and K_2CO_3 (4 g, 28.96 mmol) in degassed DMF (10 mL), 2-ethylhexylbromide (2.83 mL, 15.93 mmol) was added dropwise under nitrogen. The reaction mixture was stirred for 16 h at 80 °C. The mixture was diluted with EtOAc (50 mL), washed with H_2O (100 mL) and brine (50 mL). The organic phase was dried over $MgSO_4$ and the solvents removed under reduced pressure. The residue oil was purified by silica gel column chromatography (P.E./EtOAc in gradient 0 - 5 %), affording **1** as yellow oil (2.4 g, 91%).

^{1}H-NMR (400 MHz, $CDCl_3$) δ: 9.83 (s, 1 H), 7.41-7.38 (m, 2 H), 6.95-6.93 (d, J = 8.1 Hz, 1 H), 3.95-3.91 (m, 4 H), 1.80-1.78 (m, 2 H), 1.55-1.31 (m, 16), 0.96-0.89 (m, 12 H).
^{13}C-NMR (100 MHz, $CDCl_3$) δ: 191.22, 155.20, 149.97, 129.91, 126.76, 111.61, 110.62, 71.56, 39.60, 39.55, 29.25, 24.07, 23.21, 23.18, 14.23, 14.22, 11.34.
IMQ-QTOF-HRMS $[M + H]^+$ calc. for $[C_{23}H_{38}O_3]^+$: 362.2821, found: 362.2821.
IR (film in $CHCl_3$) ν_{max} (cm^{-1}): 2958, 2927, 2873, 2859, 1689, 1594, 1584, 1509, 1463, 1435, 1394, 1267, 1132, 1023, 808.

Synthesis of methyl 4-(di(1 H-pyrrol-2-yl)methyl)benzoate [2]

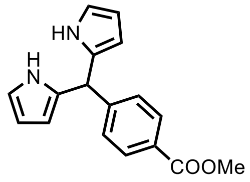

2

A solution of methyl 4-formylbenzoate (3 g, 18.29 mmol) in freshly distilled pyrrole (120 mL, 1.8 mol), was degassed with nitrogen for 45 min and protected from the light. Then TFA (150 µL, 0.18 mmol) was added dropwise and the reaction mixture was stirred at r.t. for 3 h. Subsequently a solution of NaOH (1 M) was added and the mixture was diluted with EtOAc (3 x 100 mL). The organic phase was dried over $MgSO_4$ and the solvents removed under reduced pressure. The residue was purified by silica gel column chromatography (P.E./EtOAc in gradient 5 - 70 %), affording **2** as yellow solid (3.9 g, 59 %).

^{1}H-NMR (400 MHz, C_2D_6CO) δ: 9.75 (s, 2 H), 7.92(d, J = 8.3 Hz, 2 H), 7.33 (d, J = 8.3 Hz, 2 H), 6.70-6.69 (m, 2 H), 6.00-5.98 (m, 2 H), 5.74 (s, 2 H), 5.54 (s, 1 H). 3.86 (s, 3 H).
^{13}C-NMR (100 MHz, C_2D_6CO) δ:167.15, 149.97, 133.21, 133.06, 130.07, 129.42, 129.21, 118.07, 117.90, 108.26, 108.21, 107.63, 107.61, 52.21, 44.86, 44.83.
HRMS calc. for $[C_{17}H_{17}N_2O_2]^+$: 281.1285, found: 281.1286.
IR (film in $CHCl_3$) ν_{max} (cm^{-1}): 3370, 3100, 2950, 1702, 1609, 1562, 1434, 1415, 1309, 1279, 1178, 1110, 1027, 965, 868, 813, 792, 740, 718, 544, 488.

Synthesis of 5, 15-bis(2-ethylhexoxy)phenyl-10, 20-bis(4-methoxycarbonylphenyl)-21H, 23H-porphine [3]

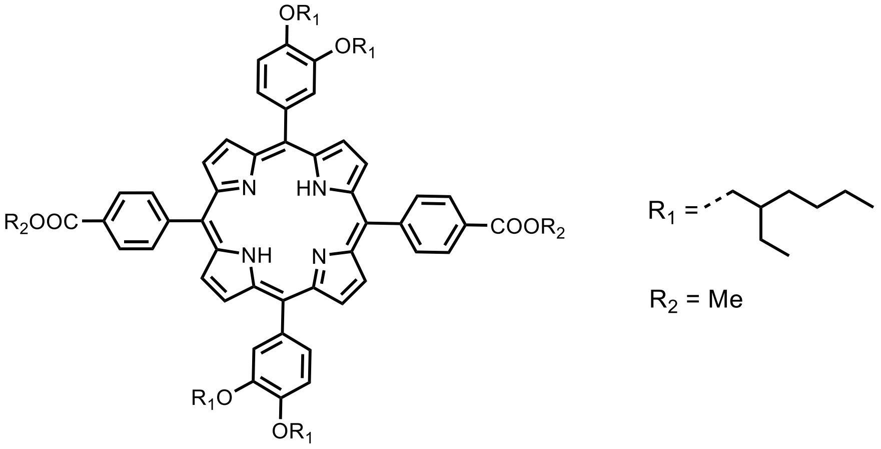

3

In a solution of **1** (514 mg, 1.42 mmol) in dry CH_2Cl_2 (300 mL), **2** (400 mg, 1.42 mmol) was added. The solution was deoxygenated by bubbling nitrogen for 1 h. then TFA (50 μL) was added under nitrogen and the reaction mixture was stirred at r.t. for 16 h, covered from the light. Subsequently DDQ (1 g, 4.26 mmol) was added and the reaction mixture was stirred for 2 h. Then NEt_3 (1 mL) was added and the solution was stirred for 1 h. The solvents were removed under reduced pressure and passed over silica plug (CH_2Cl_2) to obtain a dark purple solid. The residue was purified by silica gel column chromatography (P.E./CH_2Cl_2 1:1) affording the desired porphyrin **3** as purple solid (80 mg, 6 %).

M.P.: Foam.

^{1}H-NMR (400 MHz, $CDCl_3$) δ: 8.96 (d, J = 4.7 Hz, 4 H), 8.79 (d, J = 4.8 Hz, 4 H), 8.44 (d, J = 8.1 Hz, 4 H), 8.30 (d, J = 8.1 Hz, 4 H), 7.77 (s, 2 H), 7.71-7.69 (m, 2 H), 7.26-7.24 (m, 2 H), 4.18-4.17 (m, 4 H), 4.12 (s, 6 H), 4.01-3.99 (m, 4 H), 1.98-1.30 (m, 36 H), 1.11-0.84 (m, 24 H), -2.76 (s, 2 H) .

^{13}C-NMR (100 MHz, $CDCl_3$) δ: 167.63, 149.83, 147.84, 147.34, 134.84, 134.72, 129.90, 128.25, 127.91, 121.29, 120.98, 118.79, 111.88, 77.65, 77.33, 77.01, 72.31, 72.14, 52.75, 40.18, 40.05, 31.14, 31.14, 30.98, 30.97, 29.65, 29.50, 24.46, 24.45, 24.29, 24.28, 23.51, 23.38, 14.52, 14.39, 11.72, 11.58.

HRMS calc. for $[C_{80}H_{98}N_4O_8]^+$: 1243.7457, found: 1243.7457.

IR (film in $CHCl_3$) ν_{max} (cm^{-1}): 3350, 2956, 2925, 2871, 2858, 1724, 1606, 1510, 1466, 1273, 1259, 1133, 1111, 1021, 994, 972, 929, 800, 761, 733, 711.

Synthesis of 5, 15-bis(2-ethylhexoxy)phenyl-10, 20-bis(4-hydroxycarbonylphenyl)-21H, 23H-porphine [4]

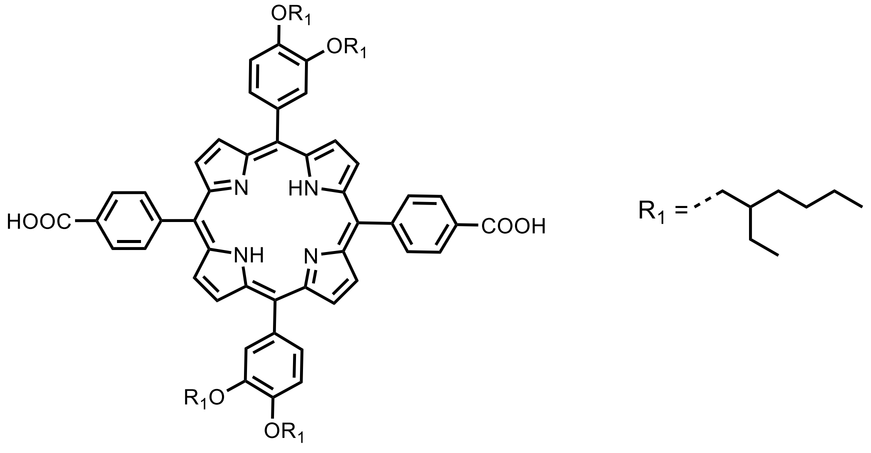

4

To a suspension of **3** (100 mg, 0.08 mmol) in EtOH (10 mL)/THF (10 mL), a solution of KOH_{aq}. 2 M (4 ml) was added. The reaction mixture was stirred at 80 °C for 16 h. The solvents were removed under reduced pressure and then a solution of 3 M HCl was added until pH 2 was achieved. The mixture was diluted with EtOAc (20 mL) and water (20 mL) and extracted (3 x 20 mL). The organic phase was dried over $MgSO_4$, and the solvents removed under reduced pressure affording the desired porphyrin **4** as purple solid (96 mg, 98 %).

M.P.: Foam.

^{1}H-NMR δ: (400 MHz, THF-*d*$_8$) δ: 8.96 (d, J = 3.9 Hz, 4 H), 8.80 (d, J = 4.1 Hz, 4 H), 8.45 (d, J = 8.0 Hz, 4 H), 8.31 (d, J = 7.6 Hz, 4 H), 7.81 (s, 2 H), 7.70 (d, J = 7.9 Hz, 2 H), 7.31 (d, J = 8.1 Hz, 2 H), 4.18-4.17 (m, 4 H), 4.09 (s,4 H), 1.94-1.89 (m, 2 H), 1.81-1.79 (m, 3 H), 1.59-1.44 (m, 20 H), 1.36-1.29 (m, 11 H), 1.10 (t, J = 7.4 Hz. 6 H), 1.01 (t, J = 7.1 Hz. 6 H), 0.96 (t, J = 7.4 Hz. 6 H), 0.87 (t, J = 7.0 Hz. 6 H), 1.11-0.84 (m, 24 H), -2.68 (s, 2 H) .

^{13}C-NMR (100 MHz, THF-*d*$_8$) δ: 167.95, 150.80, 148.92, 147.83, 135.48, 135.35, 131.61, 129.08, 128.28, 121.72, 121.36, 119.98, 112.32, 72.34, 72.26, 41.20, 40.78, 31.98, 31.47, 30.67, 30.39, 30.02, 24.50, 23.81, 15.11, 14.60, 12.35, 11.92. Some quaternary ^{13}C NMR signals are missing.

HRMS calc. for $[C_{78}H_{95}N_4O_8]^+$: 1215.7156, found: 1215.7144.

IR (film in $CHCl_3$) ν_{max} (cm^{-1}): 3443, 2956, 2925, 2870, 2857, 1687, 1604, 1508, 1464, 1411, 1379, 1254, 1223, 1133, 1019, 971, 926, 868, 797, 730, 709

Synthesis of N-(4-azidophenyl)-N-benzyl-4-((tert-butyldimethylsilyl)oxy)benzamide [6]

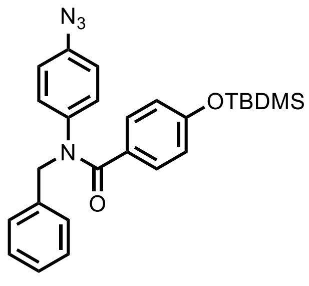

6

To a solution of **5** (500 mg, 1.35 mmol) in dry THF (10 mL) at 0 ^{0}C, a solution of NaH (110 mg, 2.7 mmol) in dry THF (10 mL) was added dropwise. The reaction mixture was stirred at 0 ^{o}C for 15 min, then BnBr (320 µL, 2.7 mmol) was added dropwise and the reaction mixture was stirred at room temperature for 16h. The reaction mixture was quenched with a sat. solution of NH_4Cl (10 mL) and then diluted with EtOAc (20 mL) and water (20 mL) and extracted (3 x 20 mL). The organic phase was dried over $MgSO_4$, and the solvents removed under reduced pressure. The residue was purified by silica gel column chromatography (P.E. / EtOAc 9-1) to afford the desired porphyrin **6** as yellow oil (153 mg, 25 %).

^{1}H-NMR (400 MHz, $CDCl_3$) δ: 7.32-7.30 (m, 4 H), 7.28-7.24 (m, 3 H), 6.89 (d, J = 8.7 Hz, 2 H), 6.81 (d, J = 8.7 Hz, 2 H), 6.65 (d, J = 8.6 Hz, 2 H), 5.12 (s, 2 H), 0.95 (s, 9 H), 0.16 (s, 6 H).

^{13}C-NMR (100 MHz, $CDCl_3$) δ: 170.36, 157.29, 140.83, 138.26, 137.61, 130.89, 129.07, 128.68, 128.65, 128.57, 128.53, 128.43, 127.55, 119.60, 119.60, 77.48, 77.16, 76.84, 53.97, 25.73, 18.35, -4.31.

HRMS calc. for $[C_{26}H_{30}N_4O_2Si]^+$: 458.2138, found: 458.2132.

IR (film in $CHCl_3$) ν_{max} (cm^{-1}): 3060, 3031, 2956, 2929, 2896, 2857, 2123, 2091, 1643, 1601, 1505, 1410, 1378, 1319, 1291, 1265, 1167, 1108, 972, 909, 837, 805, 782, 764, 726, 698, 623.

Synthesis of N-(4-azidophenyl)-N-benzyl-4-hydroxybenzamide [7]

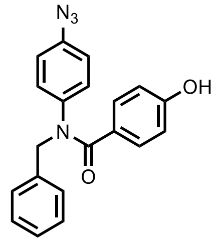

7

To a solution of **6** (140 mg, 0.305 mmol) in THF (5 mL), a solution of TBAF 1 M (610 μL, 0.61 mmol) was added dropwise at 0 °C. The reaction mixture was stirred for 15 min at room temperature. The reaction mixture was quenched with 1 M HCl until pH 2 was obtained. Then the solution was diluted with EtOAc (20 mL) and water (20 mL) and extracted (3 x 20 mL). The organic phase was dried over $MgSO_4$, and the solvents removed under reduced pressure. The residue was purified by silica gel column chromatography (P.E. / EtOAc 6-4) to afford **7** as yellow solid (102 mg, 97 %).

M.P.: 130.3-132.5 °C
^{1}H-NMR (400 MHz, $CDCl_3$) δ: 7.28 (s, 5 H), 7.18 (d, J = 8.6 Hz, 2 H), 6.90 (d, J = 8.7 Hz, 2 H), 6.82 (d, J = 8.7 Hz, 2 H), 6.58 (d, J = 8.6 Hz, 2 H), 5.10 (s, 2 H).
^{13}C-NMR (100 MHz, $CDCl_3$) δ: 171.05, 158.25, 140.52, 138.48, 137.25, 131.11, 131.05, 129.83, 129.21, 129.06, 128.68, 128.60, 128.53, 128.43, 127.65, 127.19, 126.77, 119.72, 115.04, 77.48, 77.16, 76.84, 54.26.
HRMS calc. for $[C_{20}H_{17}N_4O_2]^+$: 345.1352, found: 345.1349.
IR (film in $CHCl_3$) ν_{max} (cm^{-1}): 3284, 3062, 3031, 2955, 2122, 2093, 1608, 1577, 1504, 1434, 1392, 1321, 1295, 1277, 1228, 1170, 1110, 971, 841, 762, 726, 699.

Synthesis of Porphyrin [10-H_2]

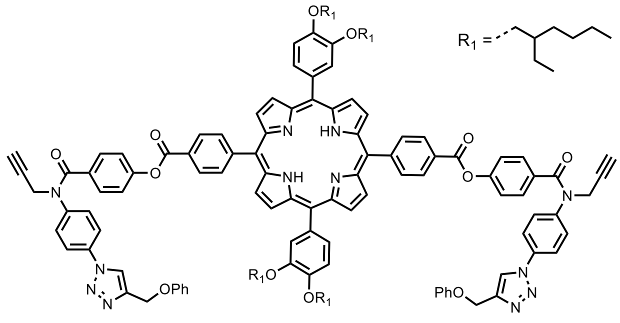

10 - H_2

A solution of porphyrin **4** (54 mg, 0.044 mmol), phenol **9** (56 mg, 0.133 mmol), EDC·HCl (50 mg, 0.264 mmol), HOBt (36 mg, 0.264 mmol), DIPEA (33 μL, 0.198 mmol) in DMF (10 mL), was stirred at room temperature for 16h. Then the solution was diluted with EtOAc (50 mL) and water (50 mL, containing 5% wt LiCl) and extracted (3 x 50 mL). The organic phase was dried over $MgSO_4$, and the solvents removed under reduced pressure. The residue was purified by silica gel column chromatography (DCM / EtOAc 95:5) to afford porphyrin **10-H_2** as a purple solid (83 mg, 94 %).

M.P.: foam.

^{1}H-NMR (400 MHz, $CDCl_3$) δ: 8.97-8.96 (m, 4 H), 8.79-8.78 (m, 4 H), 8.54 (d, J = 8.0 Hz, 4 H), 8.35 (d, J = 8.1 Hz, 4 H), 8.06 (s, 2 H), 7.76-7.69 (m, 8 H), 7.57 (d, J = 8.2 Hz, 4 H), 7.39 (d, J = 8.7 Hz, 4 H), 7.32-7.23 (m, 10 H), 7.03-6.96 (m, 6 H), 5.31 (s, 4 H), 4.78 (d, J = 2.1 Hz, 4), 4.17 (d, J = 5.5 Hz, 4 H), 3.99 (d, J = 4.6 Hz, 4 H), 2.33 (t, J = 2.3 Hz, 2 H), 1.97-1.95 (m, 2 H), 1.83-1.81 (m, 2 H), 1.72-1.26 (m, 32 H), 1.09 (t, J = 7.4 Hz, 6 H), 0.99 (t, J = 6.8 Hz, 6 H), 0.93 (t, J = 7.2 Hz, 6 H), 0.85 (t, J = 6.3 Hz, 6 H), -2.76 (s, 2 H)

^{13}C-NMR (100 MHz, $CDCl_3$) δ: 169.30, 164.87, 158.21, 152.69, 149.70, 148.20, 148.15, 147.67, 145.57, 143.30, 135.71, 134.87, 134.43, 134.37, 132.40, 130.75, 129.76, 129.10, 128.67, 128.65, 127.76, 121.73, 121.57, 121.51, 121.33, 120.99, 120.86, 120.79, 118.60, 118.23, 114.88, 111.65, 78.70, 77.41, 77.16, 76.91, 73.09, 72.17, 71.91, 62.07, 40.12, 40.00, 39.87, 30.96, 30.95, 30.79, 30.76, 29.88, 29.47, 29.32, 24.28, 24.24, 24.11, 24.10, 23.33, 23.20, 14.35, 14.22, 11.54, 11.40.

HRMS calc. for $[C_{128}H_{131}N_{12}O_{12}]^+$: 2028.0004, found: 2028.0078.

IR (film in $CHCl_3$) ν_{max} (cm^{-1}): 3307, 2958, 2927, 2874, 2857, 2251, 1737, 1650, 1602, 1497, 1466, 1378, 1349, 1259, 1246, 1203, 1069, 1018, 903, 723, 669, 648.

Synthesis of Porphyrin [10]

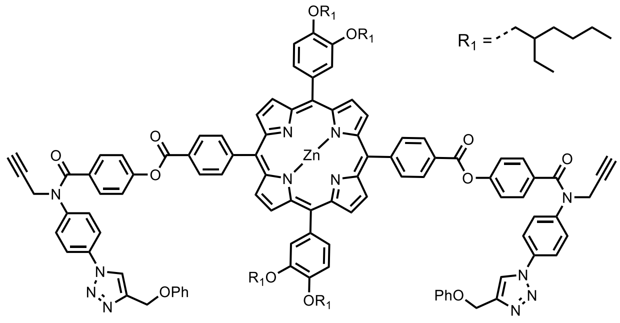

10

Porphyrin **10-H2** (83 mg, 0.041 mmol) and $Zn(OAc)_2$ (75 mg, 0.41 mmol) were dissolved in dry $CHCl_3$ (10 mL). The reaction mixture was stirred at room temperature for 16 h. The solution was concentrated under reduced pressure, and the residue was triturated with MeOH. The purple solid was filtered and washed with MeOH to afford porphyrin **10** as purple solid (83 mg, 97 %).

M.P.: foam.

^{1}H-NMR (400 MHz, $CDCl_3$) δ: 9.06-9.04 (m, 4 H), 8.86-8.84 (m, 4 H), 8.49 (d, J = 7.4 Hz, 4 H), 8.32 (d, J = 7.4 Hz, 4 H), 7.79-7.77 (m, 2 H), 7.68 (m, 2 H), 7.61 (m, 2 H), 7.45-7.3 (m, 8 H), 7.26-7.22 (m, 14 H), 6.93-6.92 (m, 2H), 6.73-6.69 (m, 4 H), 4.64 (s, 4 H), 4.29-4.18 (m, 8 H), 3.96-3.94 (m, 4 H), 2.29 (s, 2 H), 1.96-1.94 (m, 2 H), 1.72-1.26 (m, 32 H), 1.09 (t, J = 7.4 Hz, 6 H), 0.99 (t, J = 6.9 Hz, 6 H), 0.92 (t, J = 7.3 Hz, 6 H), 0.85 (t, J = 6.3 Hz, 6 H).

^{13}C-NMR (100 MHz, $CDCl_3$) δ: 169.17, 165.00, 157.81, 157.79, 152.68, 152.67, 151.03, 150.91, 150.70, 149.75, 149.54, 149.43, 149.40, 149.33, 149.31, 147.56, 147.51, 144.64, 144.60, 143.22, 135.51, 135.22, 134.95, 134.90, 132.77, 132.65, 132.45, 132.20, 131.64, 131.44, 131.31, 130.65, 129.75, 129.68, 129.67, 128.94, 128.39, 128.15, 127.73, 122.02, 121.71, 121.52, 121.51, 121.08, 120.96, 119.99, 119.37, 119.10, 114.83, 114.65, 114.64, 111.58, 78.59, 77.41, 77.16, 76.91, 73.09, 72.07, 72.01, 60.91, 40.03, 39.89, 30.96, 30.78, 29.48, 29.32, 24.28, 24.27, 24.10, 24.08, 23.33, 23.20, 14.35, 14.22, 11.56, 11.41.

MS calc. for $[C_{128}H_{129}N_{12}O_{12}Zn]^{+}$: 2089.9, found: 2089.8; calc. for $[C_{128}H_{130}N_{12}O_{12}Zn]^{2+}$: 1045.5, found 1045.3.

IR (film in $CHCl_3$) ν_{max} (cm^{-1}): 3301, 3291, 3273, 3064, 2956, 2927, 2871, 2859, 1739, 1656, 1601, 1518, 1494, 1462, 1409, 1376, 1338, 1257, 1203, 1165, 1135, 1066, 1032, 1012, 996, 873, 796, 755, 732, 720, 632.

Synthesis of Porphyrin [11-H_2]

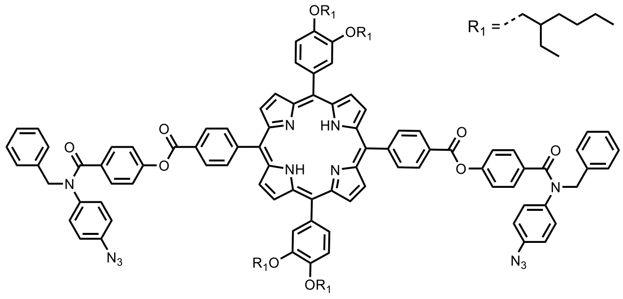

11-H2

A solution of porphyrin **4** (54 mg, 0.044 mmol), phenol **7** (45 mg, 0.133 mmol), EDC·HCl (50 mg, 0.264 mmol), HOBt (36 mg, 0.264 mmol), DIPEA (33 μL, 0.198 mmol) in DMF (10 mL), was stirred at room temperature for 16h. Then the solution was diluted with EtOAc (50 mL) and water (50 mL, containing 5% wt LiCl) and extracted (3 x 50 mL). The organic phase was dried over $MgSO_4$, and the solvents removed under reduced pressure. The residue was purified by silica gel column chromatography (DCM) to afford porphyrin **11-H$_2$** as a purple solid (65 mg, 79 %).

M.P.: foam.

^{1}H-NMR (400 MHz, $CDCl_3$) δ: 8.97 (d, J = 4.7 Hz, 4 H), 8.79 (d, J = 4.8 Hz, 4 H), 8.55 (d, J = 8.1 Hz, 4 H), 8.36 (d, J = 8.1 Hz, 4 H), 7.76 (s, 2 H), 7.70 (d, J = 8.0 Hz, 2 H), 7.52 (d, J = 8.55 Hz, 4 H), 7.34-7.24 (m, 16 H), 6.96 (d, J = 8.7 Hz, 4 H), 6.88 (d, J = 8.7 Hz, 4 H), 5.15 (s, 4 H), 4.17 (d, J = 5.7 Hz, 4), 3.99 (d, J = 5.9 Hz, 4 H), 1.96-1.94 (m, 2 H), 1.83-1.81 (m, 2 H), 1.54-1.26 (m, 32 H), 1.08 (t, J = 7.5 Hz, 6 H), 0.99 (t, J = 7 Hz, 6 H), 0.93 (t, J = 7.4 Hz, 6 H), 0.85 (t, J = 6.9 Hz, 6 H), -2.76 (s, 2 H).

^{13}C-NMR (100 MHz, $CDCl_3$) δ: 169.64, 168.94, 164.94, 152.19, 151.75, 149.69, 148.09, 147.67, 140.26, 140.20, 138.80, 138.69, 137.33, 137.28, 134.88, 134.45, 133.52, 133.24, 130.69, 130.61, 130.36, 129.29, 129.19, 128.75, 128.70, 128.68, 128.61, 127.76, 127.71, 121.41, 121.30, 121.12, 120.81, 119.85, 119.82, 118.28, 111.73, 77.41, 77.16, 76.91, 72.09, 71.95, 54.17, 40.00, 39.87, 30.95, 30.79, 29.83, 29.45, 29.32, 24.30, 24.10, 23.35, 23.15, 14.35, 14.15, 11.54, 11.35.

HRMS calc. for $[C_{118}H_{123}N_{12}O_{10}]^+$: 1867.9485, found: 1867.9496.

IR (film in $CHCl_3$) ν_{max} (cm^{-1}): 3193, 3062, 2955, 2923, 2915, 2871, 2857, 2128, 2118, 2094, 2085, 1739, 1646, 1601, 1504, 1466, 1377, 1294, 1257, 1199, 1165, 1133, 1066, 1017, 971, 927, 825, 812.

Synthesis of Porphyrin [11]

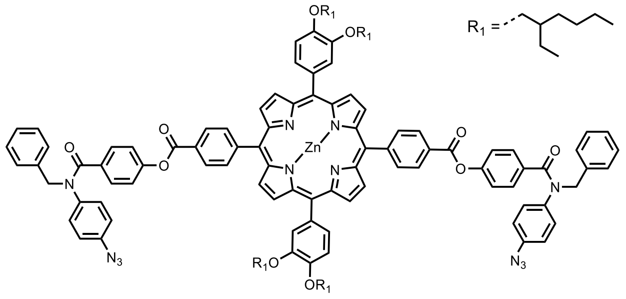

11

Porphyrin **11-H_2** (57 mg, 0.035 mmol) and $Zn(OAc)_2$ (64 mg, 0.35 mmol) were dissolved in dry $CHCl_3$ (5 mL). The reaction mixture was stirred at room temperature for 16 h. The solution was concentrated under reduced pressure, and the residue was triturated with MeOH. The purple solid was filtered and washed with MeOH to afford porphyrin **11** as purple solid (46 mg, 68 %).

M.P.: foam.

^{1}H-NMR (400 MHz, $CDCl_3$) δ: 9.08 (d, J = 4.5 Hz, 4 H), 8.87 (d, J = 4.4 Hz, 4 H), 8.53 (d, J = 7.8 Hz, 4 H), 8.32 (d, J = 7.0 Hz, 4 H), 7.80-7.72 (m, 4 H), 7.26 (s, 6 H), 7.18-7.311 (m, 14 H), 6.82 (s, 8H), 4.79 (d, J = 5.4 Hz, 4 H), 4.19 (d, J = 5.0 Hz 4 H), 4.01 (s, 4 H), 1.97 (m, 2 H), 1.83-1.82 (m, 2 H), 1.74-1.26 (m, 32 H), 1.09 (t, J = 7.4 Hz, 6 H), 0.99 (t, J = 6.9 Hz, 6 H), 0.92 (t, J = 7.3 Hz, 6 H), 0.85 (t, J = 6.3 Hz, 6 H).

^{13}C-NMR (100 MHz, $CDCl_3$) δ: 169.10, 164.97, 152.07, 150.82, 149.57, 149.47, 149.08, 147.55, 139.75, 138.78, 136.86, 135.38, 134.99, 134.91, 132.69, 132.59, 132.51, 131.52, 130.23, 129.11, 128.62, 128.42, 128.30, 127.69, 121.70, 121.22, 120.90, 119.80, 119.47, 111.61, 77.48, 77.16, 76.84, 72.14, 71.87, 53.72, 40.02, 39.89, 30.97, 30.80, 29.80, 29.48, 29.33, 24.28, 24.10, 23.34, 23.25, 14.30, 14.23, 11.57, 11.42.

HRMS calc. for $[C_{118}H_{121}N_{12}O_{10}Zn]^+$: 1929.8620, found: 1929.8643.

IR (film in $CHCl_3$) ν_{max} (cm^{-1}): 2957, 2926, 2858, 2123, 2091, 1740, 1649, 1602, 1504, 1463, 1380, 1293, 1258, 1202, 1165, 1135, 1066, 1010, 997, 796.

Synthesis of Porphyrin [12]

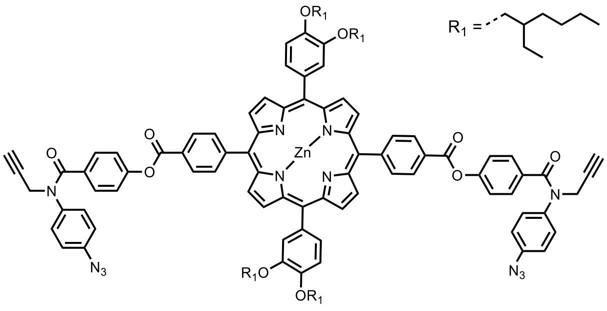

12

A solution of porphyrin **4** (60 mg, 0.049 mmol), phenol **8** (43 mg, 0.148 mmol), EDC·HCl (56 mg, 0.294 mmol), HOBt (39 mg, 0.294 mmol), DIPEA (50 µL, 0.3 mmol) in DMF (10 mL), was stirred at room temperature for 16h. Then the solution was diluted with EtOAc (50 mL) and water (50 mL, containing 5% wt LiCl) and extracted (3 x 50 mL). The organic phase was dried over $MgSO_4$, and the solvents removed under reduced pressure. The residue was purified by silica gel column chromatography (DCM / EtOAc 95:5). A solution of the resulting free base porphyrin **12-H$_2$** (55 mg, 0.031 mmol) and $Zn(OAc)_2$ (57 mg, 0.31 mmol) in dry $CHCl_3$ (5 mL) was stirred at room temperature for 16 h. The solution concentrated under reduced pressure and the residue was triturated with MeOH. The purple solid was filtered and washed with MeOH to afford porphyrin **12** as purple solid (46 mg, 87 %).

M.P.: foam.

^{1}H-NMR (400 MHz, $CDCl_3$) δ: 9.08-9.07 (m, 4 H), 8.90-8.89 (m, 4 H), 8.55 (d, J = 7.8 Hz, 4 H), 8.36 (d, J = 7.0 Hz, 4 H), 7.78 (s, 2 H), 7.72-7.70 (m, 4 H), 7.44-7.42 (m, 4 H), 7.17-7.15 (m, 8 H), 6.99-6.97 (m, 4 H), 4.58 (m, 4 H), 4.19-4.18 (m, 4 H), 3.98 (m, 4 H), 2.27 (s, 2 H), 1.97 (m, 2 H), 1.83-1.81 (m, 2 H), 1.71-1.26 (m, 32 H), 1.11-1.07 (m, 6 H), 1.00-0.98 (m, 6 H), 0.95-0.90 (m, 6 H), 0.87-0.85 (m, 6 H).

^{13}C-NMR (100 MHz, $CDCl_3$) δ: 169.25, 164.97, 152.46, 151.03, 150.88, 150.75, 149.81, 149.79, 149.60, 149.51, 148.96, 148.94, 148.85, 147.53, 139.50, 139.37, 135.15, 134.81, 132.88, 132.65, 132.39, 131.80, 131.57, 131.49, 130.65, 129.29, 128.53, 128.49, 127.54, 122.24, 121.48, 120.69, 120.09, 111.60, 78.86, 77.48, 77.16, 76.84, 72.79, 72.11, 71.96, 40.02, 39.88, 30.97, 30.79, 29.86, 29.48, 29.32, 24.28, 24.10, 23.35, 23.21, 14.37, 14.23, 11.57, 11.41.

HRMS calc. for $[C_{110}H_{113}N_{12}O_{10}Zn]^+$: 1825.7989, found: 1825.7916.

IR (film in $CHCl_3$) ν_{max} (cm^{-1}): 3302, 2956, 2927, 2855, 2122, 2095, 1738, 1649, 1602, 1494, 1460, 1378, 1349, 1259, 1244, 1210, 1065, 1010, 905, 722, 669, 650.

Synthesis of Porphyrin [13·DABCO]

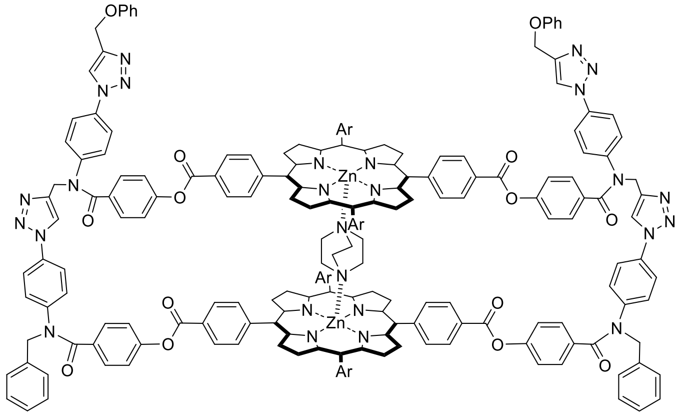

A solution of porphyrin **10** (9.5 mg, 0.00458 mmol), porphyrin **11** (8.8 mg, 0.00458 mmol) in dry CH_2Cl_2 (300 mL) was degassed for 15 min under N_2 atmosphere. Then a solution of DABCO (0.45 mg, 0.00458 mmol) in 5 mL of degassed CH_2Cl_2 was added followed by $Cu(CH_3CN)_4PF_6$ (3 mg, 0.0082 mmol) and TBTA (4.3 mg, 0.0082 mmol). The reaction mixture was stirred for 16h at room temperature. Then, the solvent was concentrated under reduce pressure and the residue was purified by gel permeation chromatography recycling mode in $CHCl_3$ to afford **13·DABCO** as a purple solid (14 mg, 79 %).

M.P.: foam.

^{1}H-NMR (400 MHz, $CDCl_3$) δ: 8.68-8.66 (m, 8 H), 8.51-8.42 (m, 14 H), 8.32-810 (m, 4 H), 8.18-810 (m, 6 H), 7.79-7.61 (m, 21 H), 7.55-7.53 (m, 6 H), 7.37-7.28 (m, 18 H), 7.27-7.22 (m, 12 H), 7.08-7.03 (m, 10 H), 5.34 (s, 4 H), 5.22 (s, 4 H), 4.21-4.08 (m, 10 H), 3.84-3.76 (m, 5 H), 1.96 (m, 4 H), 1.74-1.41 (m, 69 H), 1.15-0.78 (m, 60 H), -4.65-(-4.69) (m, 8 H).

^{13}C-NMR (100 MHz, $CDCl_3$) δ: 169.34, 169.16, 168.82, 168.80, 164.82, 164.58, 158.07, 157.72, 157.36, 152.76, 152.53, 152.44, 152.21, 150.82, 150.65, 150.49, 150.18, 150.08, 149.52, 149.39, 149.21, 149.11, 149.00, 148.93, 148.69, 148.67, 147.47, 147.33, 145.22, 144.76, 144.01, 143.78, 143.61, 136.93, 136.76, 135.34, 135.22, 135.13, 134.91, 134.76, 134.52, 134.08, 132.56, 132.48, 132.21, 132.03, 131.31, 131.17, 131.03, 130.87, 130.43, 130.28, 129.62, 129.47, 129.39, 128.77, 128.70, 128.66, 128.49, 128.45, 128.20, 128.03, 127.72, 125.64, 125.52, 125.03, 121.81, 121.41, 121.26, 120.87, 120.69, 120.35, 119.28, 118.74, 114.75, 114.51, 114.32, 111.40, 77.28, 77.02, 76.77, 61.80, 54.49, 53.57, 40.38, 40.02, 39.87, 39.72, 39.62, 38.67, 38.27, 32.35, 32.22, 31.65, 31.37, 30.81, 30.62, 30.54, 29.72, 29.59, 29.39, 29.32, 29.16, 29.07, 26.41, 26.33, 24.11, 24.01, 23.92, 23.85, 23.51, 23.45, 23.35, 23.18,

23.04, 22.95, 22.49, 14.24, 14.20, 14.06, 14.00, 13.80, 11.52, 11.47, 11.41, 11.36, 11.32, 11.25, 11.19, 11.01, 1.25, 1.04, 0.97.

HRMS MALDI $[M]^+$ calc. for $[C_{254}H_{264}N_{24}O_{22}Zn_2]^+$: 4017.7608, found: 4017.7405.

IR (film in $CHCl_3$) ν_{max} (cm^{-1}): 3080, 2955, 2930, 2871, 2849, 1745, 1650, 1601, 1518, 1454, 1460, 14010, 1378, 1336, 1255, 1203, 1165, 1135, 1066, 1030, 1012, 996, 873, 800, 750, 730, 722, 630.

Templated Mixture of Porphyrin [*p*-15·DABCO] and [*a*-15·DABCO]

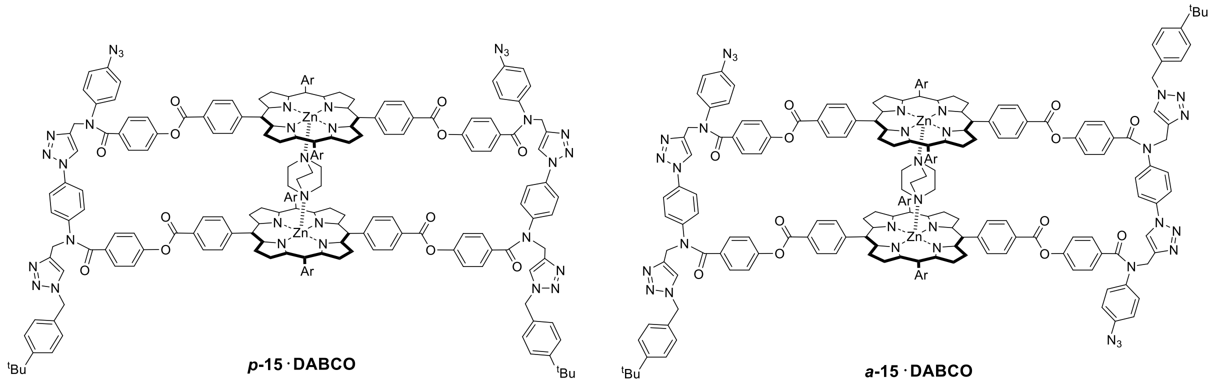

A solution of porphyrin **12** (15 mg, 0.0082 mmol) in dry CH_2Cl_2 (400 mL) was degassed for 15 min under N_2 atmosphere. A solution of DABCO (0.46 mg, 0.0041 mmol) in 5 mL of degassed CH_2Cl_2 was added to this solution, then 1-(azidomethyl)-4-(tert-butyl)benzene (31 mg, 0.164 mmol), $Cu(CH_3CN)_4PF_6$ (6 mg, 0.0164 mmol) and TBTA (8.7 mg, 0.0164 mmol) were added to the mixture. The reaction mixture was stirred for 16h at room temperature. Then, the solvent was concentrated under reduce pressure and the residue was purified by gel permeation chromatography recycling mode in $CHCl_3$ to afford the mixture ***p*-15·DABCO** and ***a*-15·DABCO** as a purple solid (8 mg, 48 %).

M.P.: foam.

^{1}H-NMR (400 MHz, $CDCl_3$) δ: 8.68-8.64 (m, 8 H), 8.51-8.43 (m, 14 H), 8.34-830 (m, 4 H), 8.19-817 (m, 4 H), 7.84-7.82 (m, 4 H), 7.78-7.70 (m, 8 H), 7.54-7.41 (m, 24 H), 7.28-7.20 (m, 12 H), 7.06-7.04 (m, 10 H), 5.57-5.52 (s, 4 H), 5.30-5.27 (s, 4 H), 5.17 (s, 4 H), 4.20-4.14 (m, 10 H), 3.97 (s, 2 H), 3.82-3.77 (m, 6 H), 1.96 (m, 4 H), 1.70-1.64 (m16 H), 1.56-1.48 (m, 35 H), 1.41-1.22 (m, 27 H), 1.13-1.11 (25 H), 0.91-0.80 (29 H), -4.61-(-4.74) (m, 8 H).

^{13}C-NMR (100 MHz, $CDCl_3$) δ: δ 168.94, 168.65, 168.28, 164.84, 164.57, 152.67, 152.57, 152.21, 151.85, 150.78, 150.65, 150.24, 150.05, 149.36, 149.28, 149.18, 148.89, 148.64, 147.48, 147.41, 147.20, 146.85, 144.75, 144.10, 141.28, 138.57, 138.49, 135.19, 135.02, 134.87, 134.69, 134.59, 134.00, 133.33, 132.65, 131.94, 131.43, 130.99, 130.88, 130.73, 130.54, 130.41, 129.53, 128.67, 128.45, 128.30, 128.10, 127.89, 127.79, 126.84, 126.62, 126.06, 126.02, 121.51, 121.33, 121.01, 120.89, 120.34, 119.94, 119.84, 118.67, 118.51, 111.32, 111.12, 77.26, 77.01, 76.76, 72.64, 72.00, 71.80, 71.67, 71.49, 53.97, 46.88, 46.81, 39.81, 39.71, 39.60, 38.62, 34.67, 31.28, 30.83, 30.64, 30.53, 29.75, 29.37, 29.33, 29.17, 29.06, 24.11, 23.94, 23.84, 23.19, 23.05, 22.96, 22.70, 14.20, 14.13, 14.07, 13.99, 11.54, 11.48, 11.42, 11.35, 11.26, 11.15, 1.04.

IR (film in $CHCl_3$) ν_{max} (cm^{-1}): 3089, 3040, 2956, 2932, 2871, 2849, 2125, 2093, 1741, 1652, 1601, 1454, 1459, 1401, 1378, 1340, 1255, 1203, 1165, 1135, 1060, 1033, 1012, 996, 873, 801, 752, 730, 721, 633.

Characterization

Characterization of 1

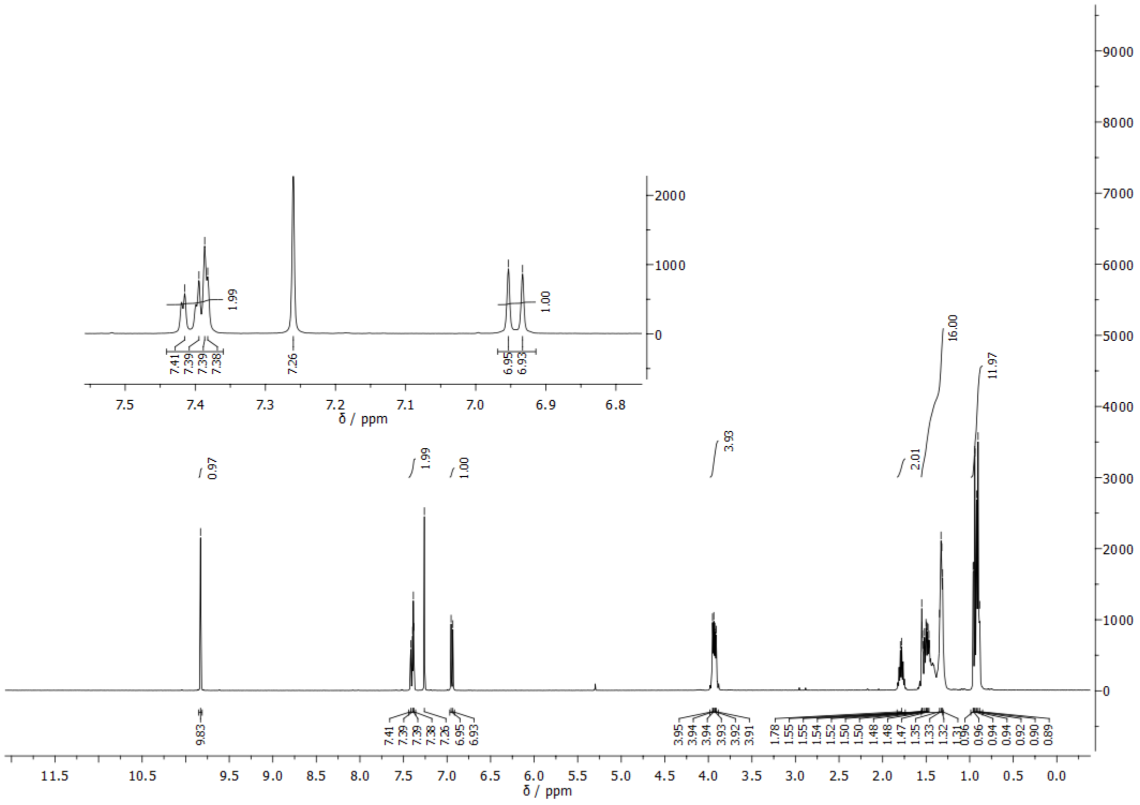

Figure 1.^{1}H-NMR, 400 MHz, $CDCl_3$.

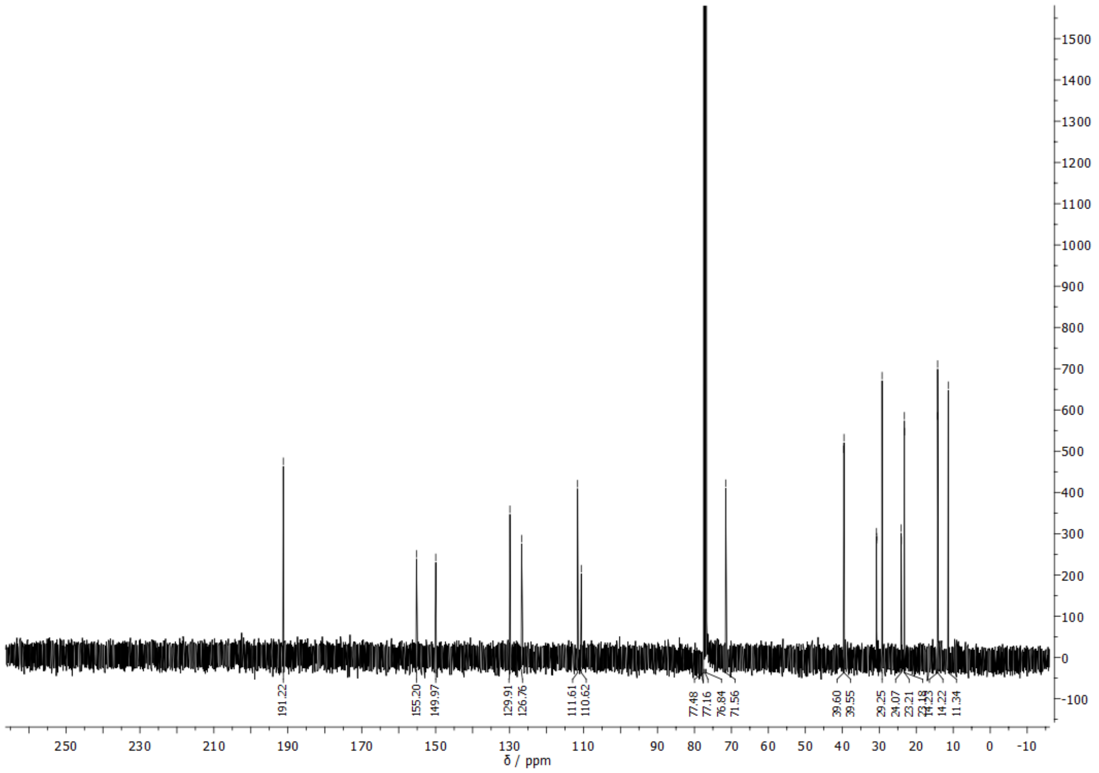

Figure 2.^{13}C-NMR, 100 MHz, $CDCl_3$.

Characterization of 2

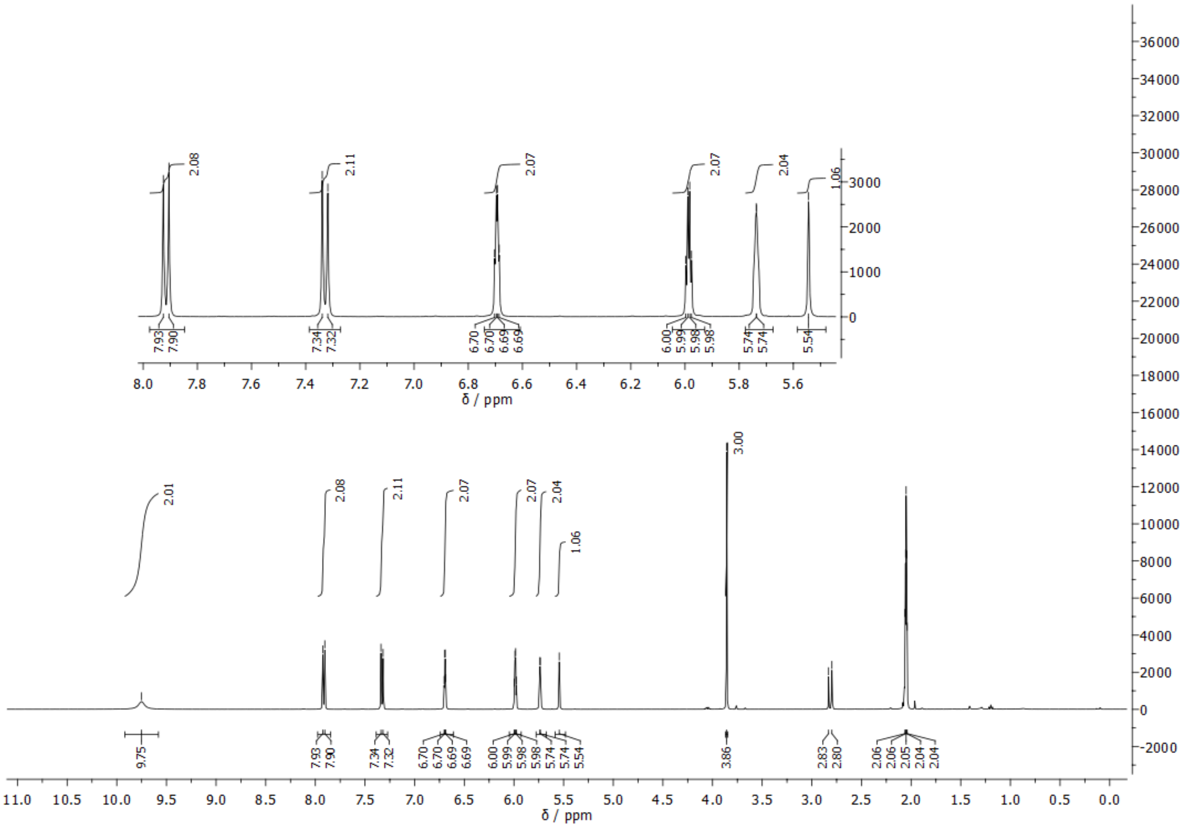

Figure 3.^{1}H-NMR, 400 MHz, C_2D_6CO.

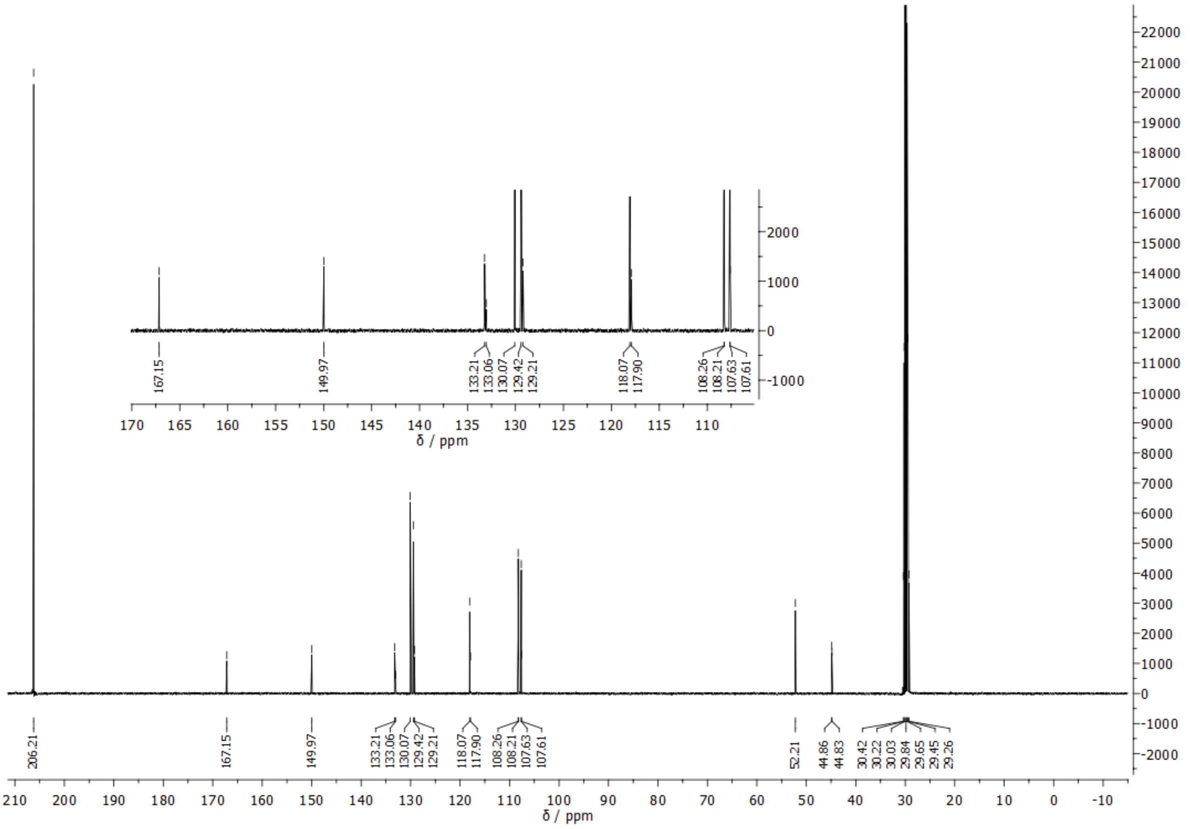

Figure 4.^{13}C-NMR, 100 MHz, C_2D_6CO.

Characterization of 3

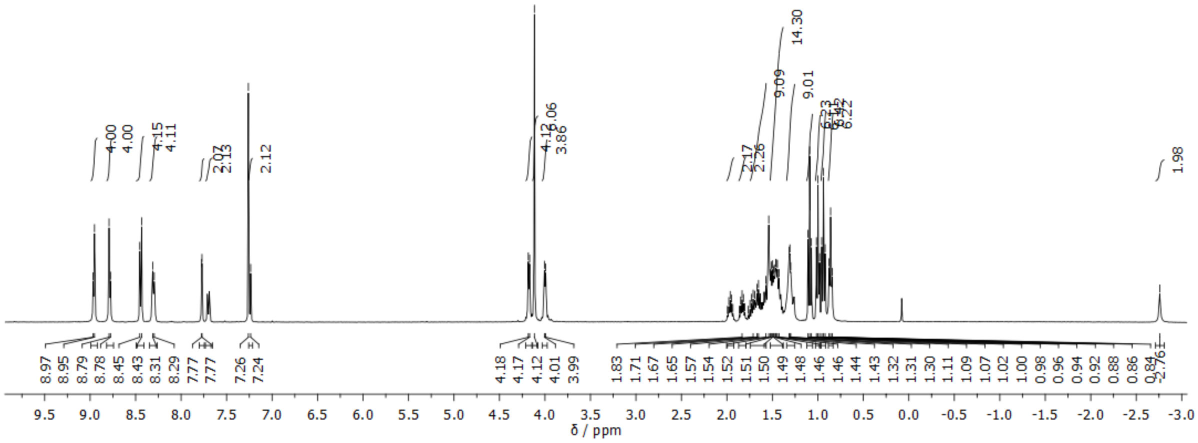

Figure 5.^{1}H-NMR, 400 MHz, $CDCl_3$.

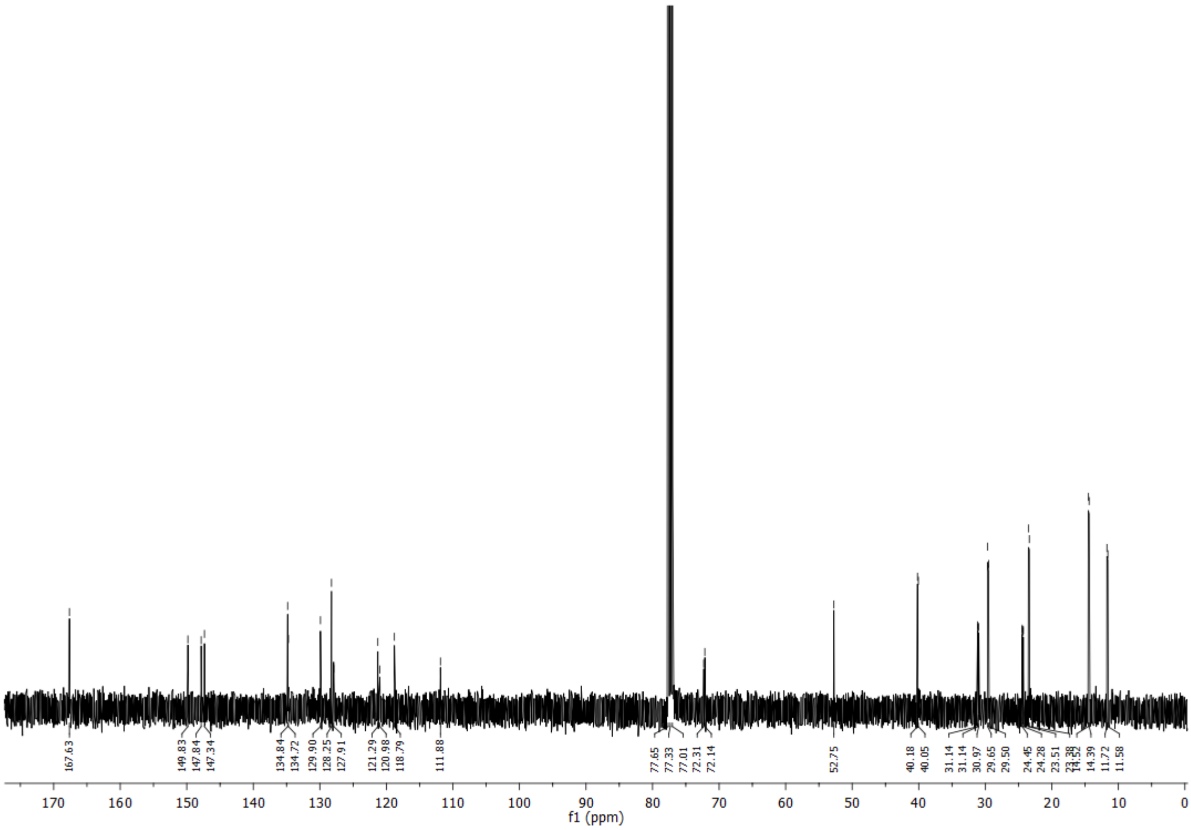

Figure 6.^{13}C-NMR, 100 MHz, $CDCl_3$.

Characterization of 4

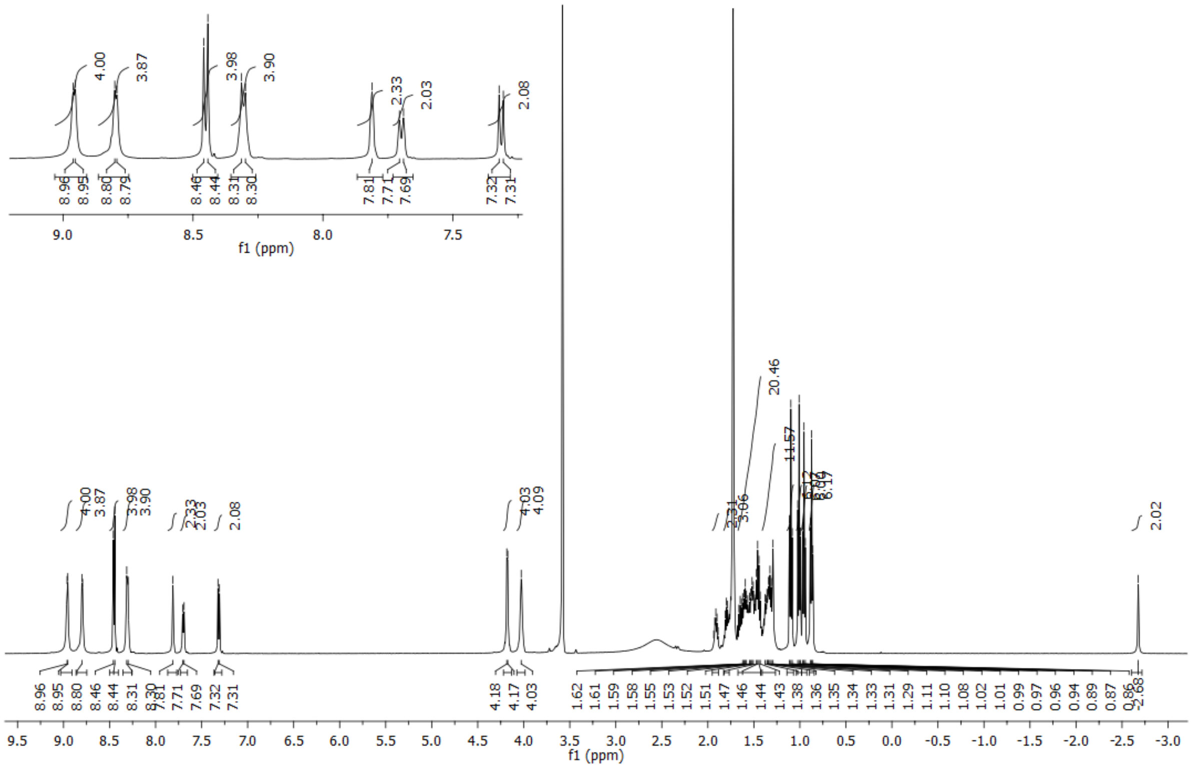

Figure 7.^{1}H-NMR, 400 MHz, THF-d_8.

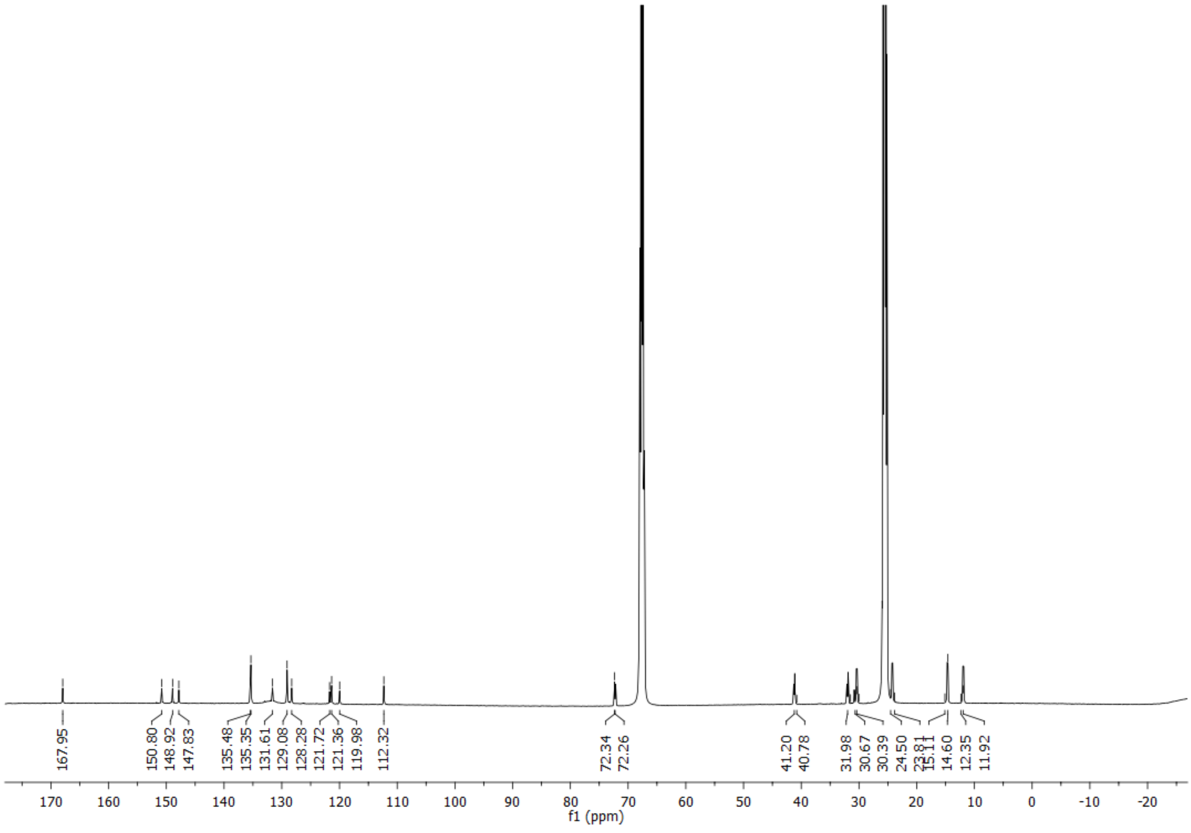

Figure 8.^{13}C-NMR, 100 MHz, THF-d_8.

Characterization of 6

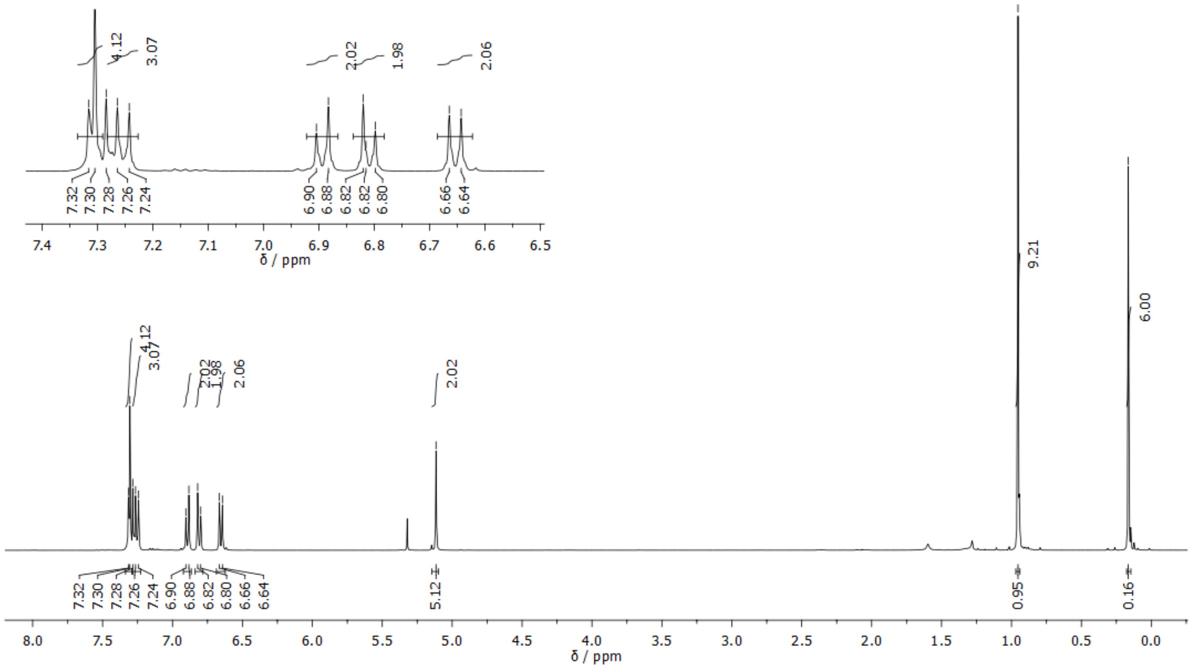

Figure 9.^{1}H-NMR, 400 MHz, CD_2Cl_2.

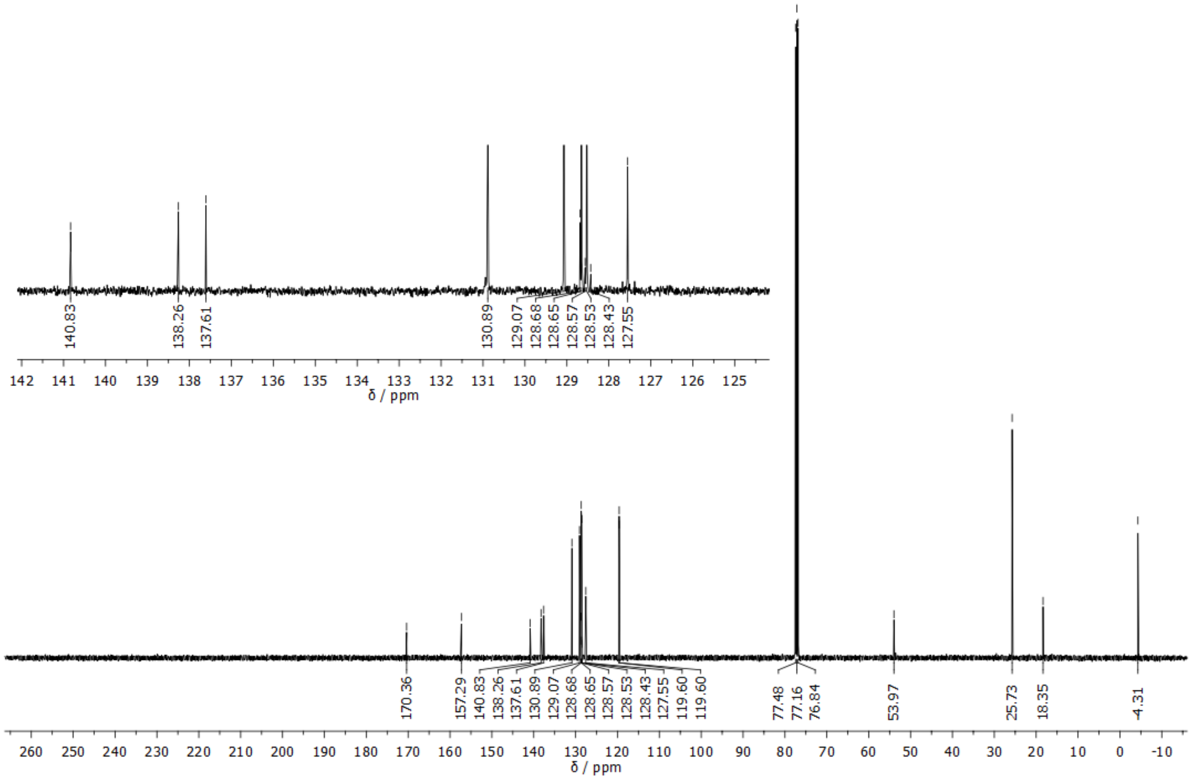

Figure 10.^{13}C-NMR, 100 MHz, $CDCl_3$.

Characterization of 7

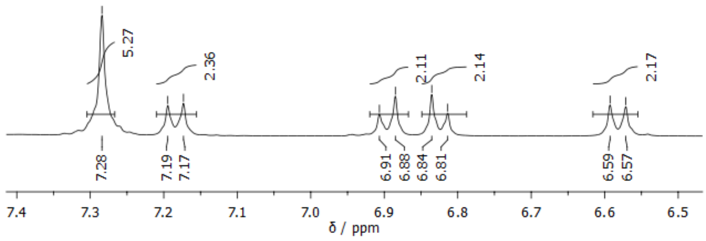

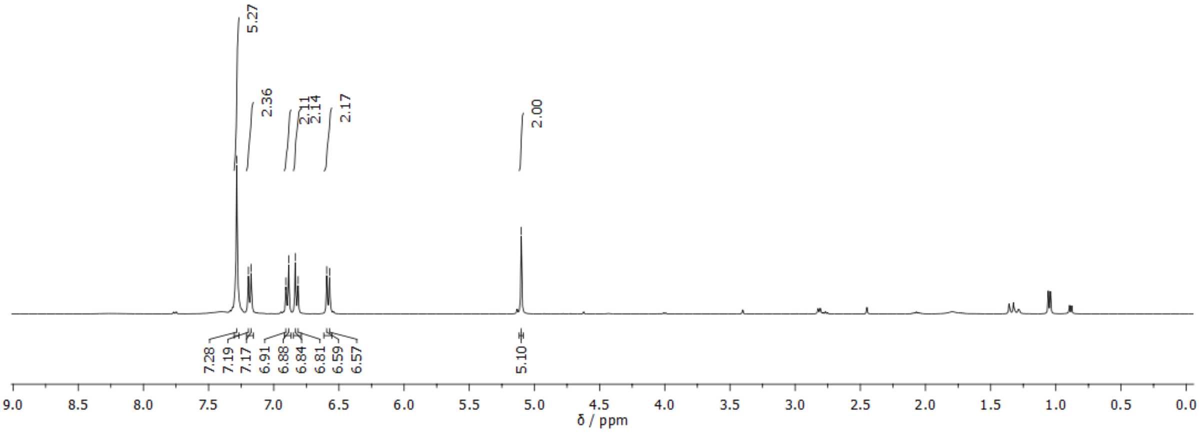

Figure 11.^{1}H-NMR, 400 MHz, $CDCl_3$.

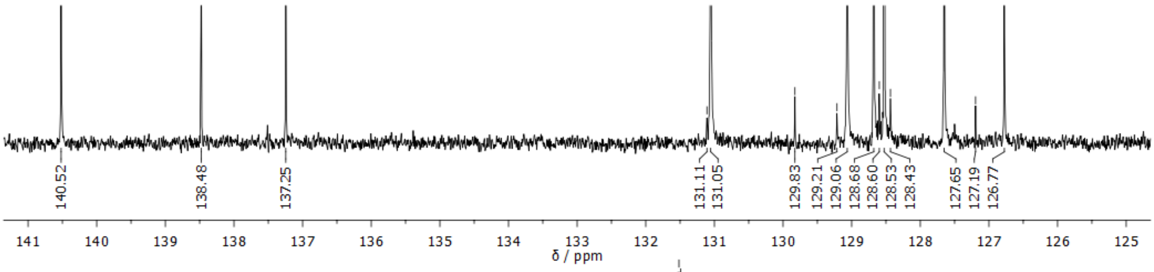

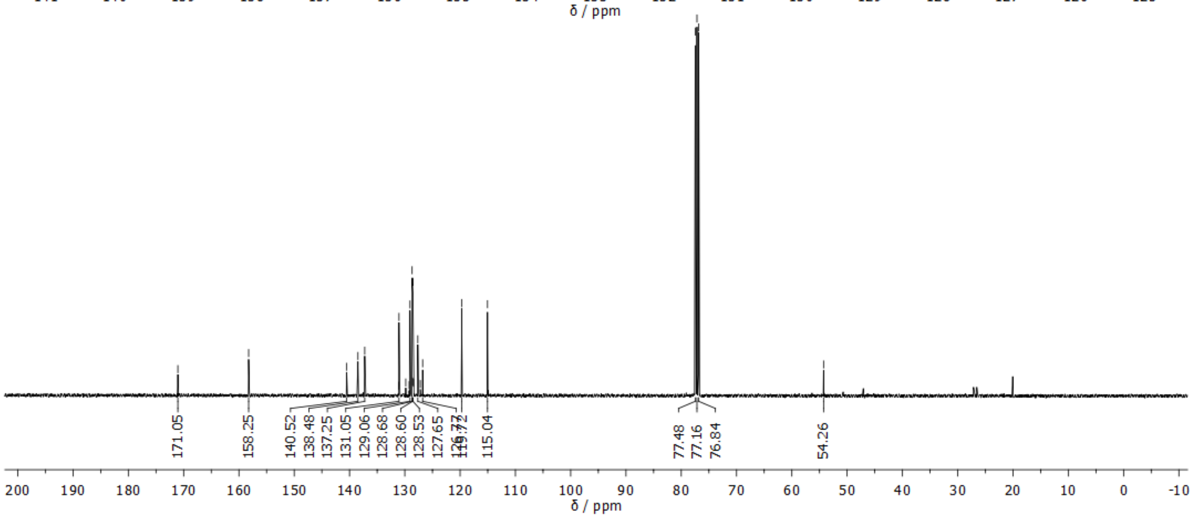

Figure 12.^{13}C-NMR, 100 MHz, $CDCl_3$.

Characterization of 10-H_2

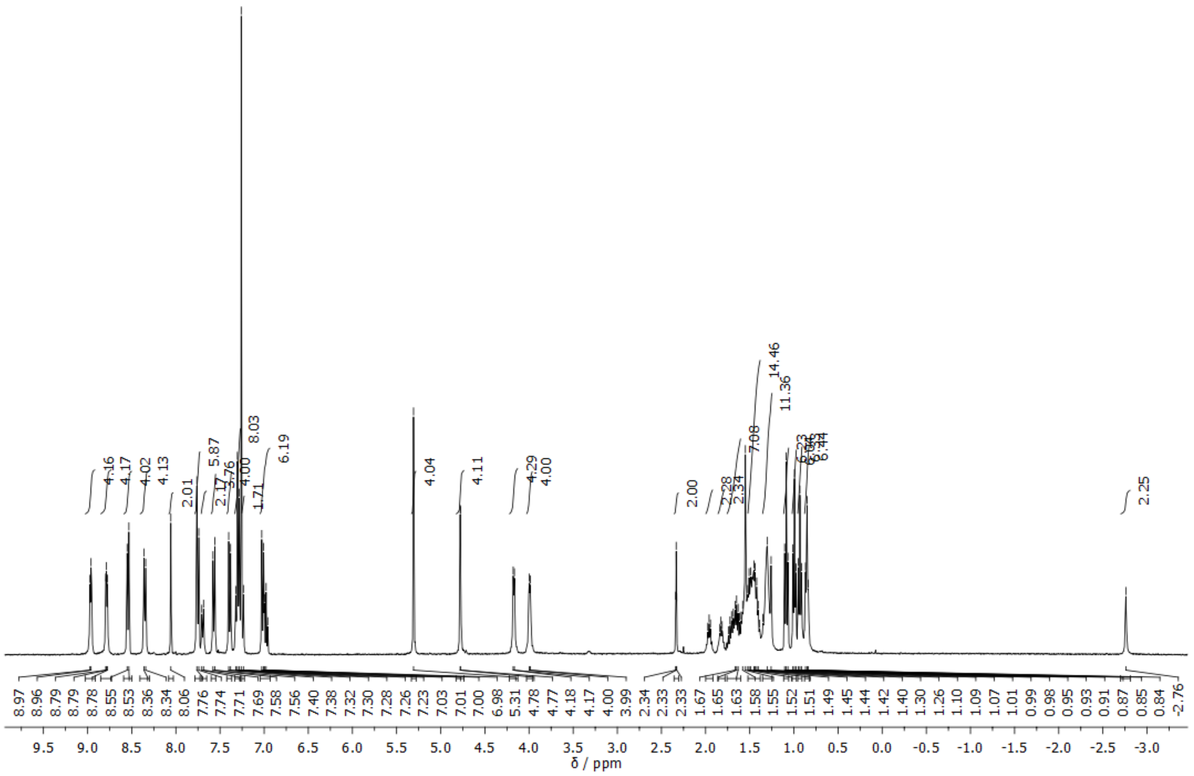

Figure 13.^{1}H-NMR, 400 MHz, $CDCl_3$.

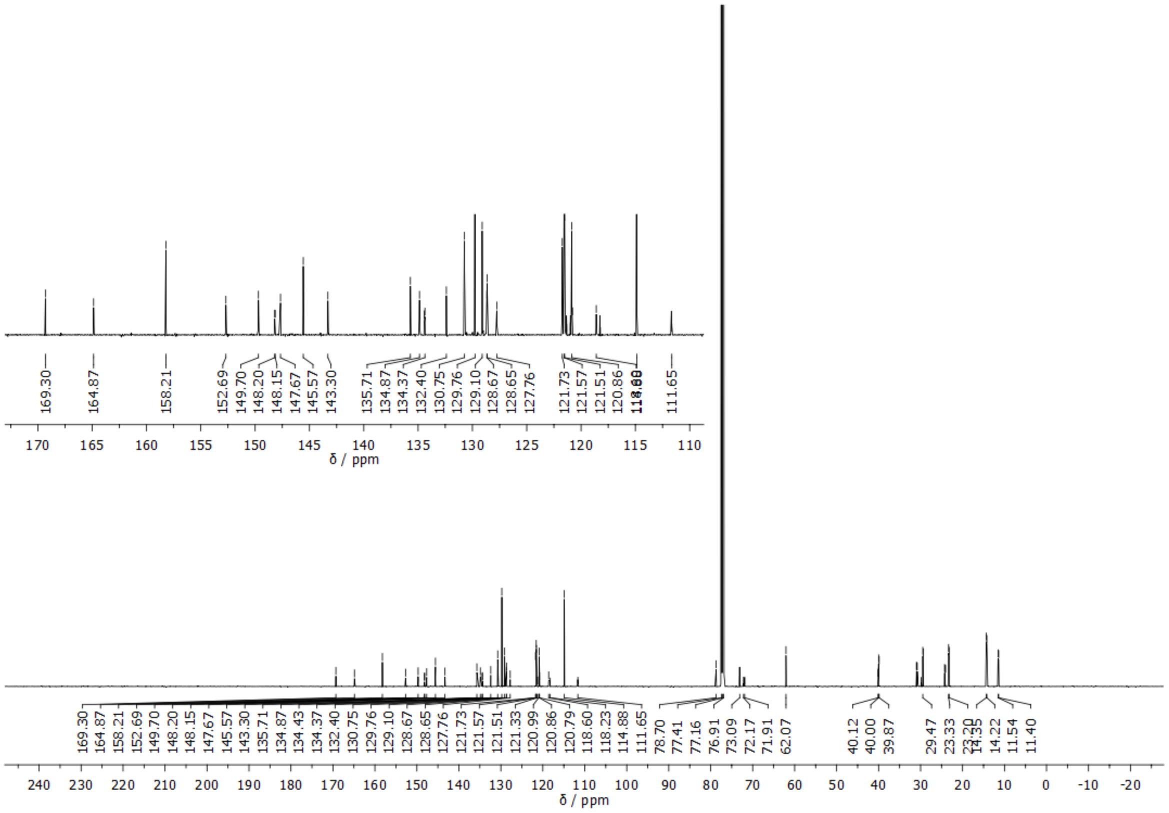

Figure 14.^{13}C-NMR, 100 MHz, $CDCl_3$.

Characterization of 10

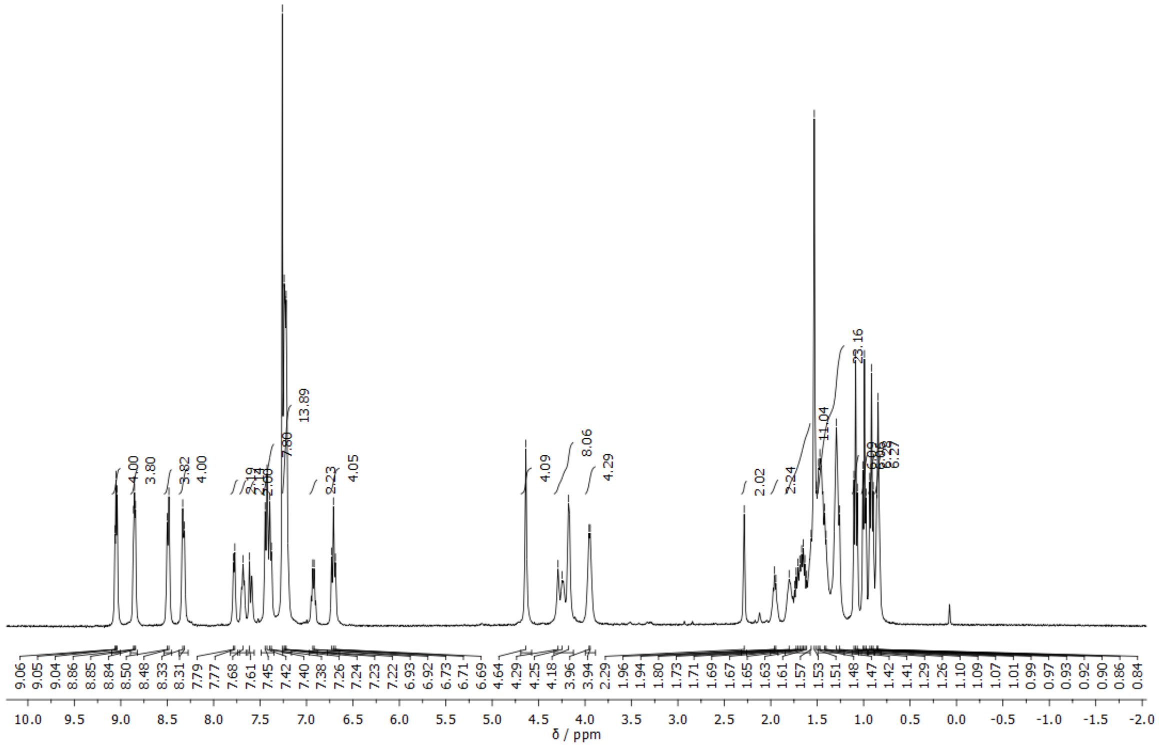

Figure 15.^{1}H-NMR, 400 MHz, $CDCl_3$.

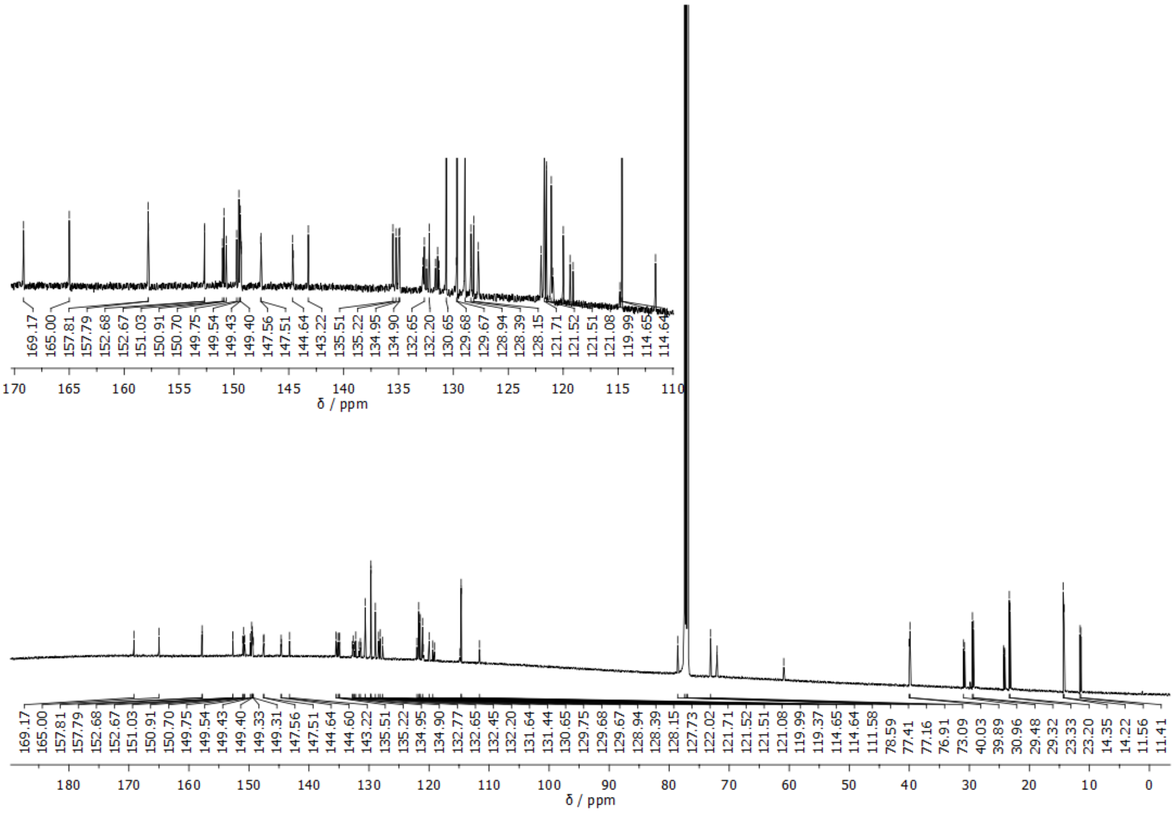

Figure 16.^{13}C-NMR, 100 MHz, $CDCl_3$.

Characterization of 11-H_2

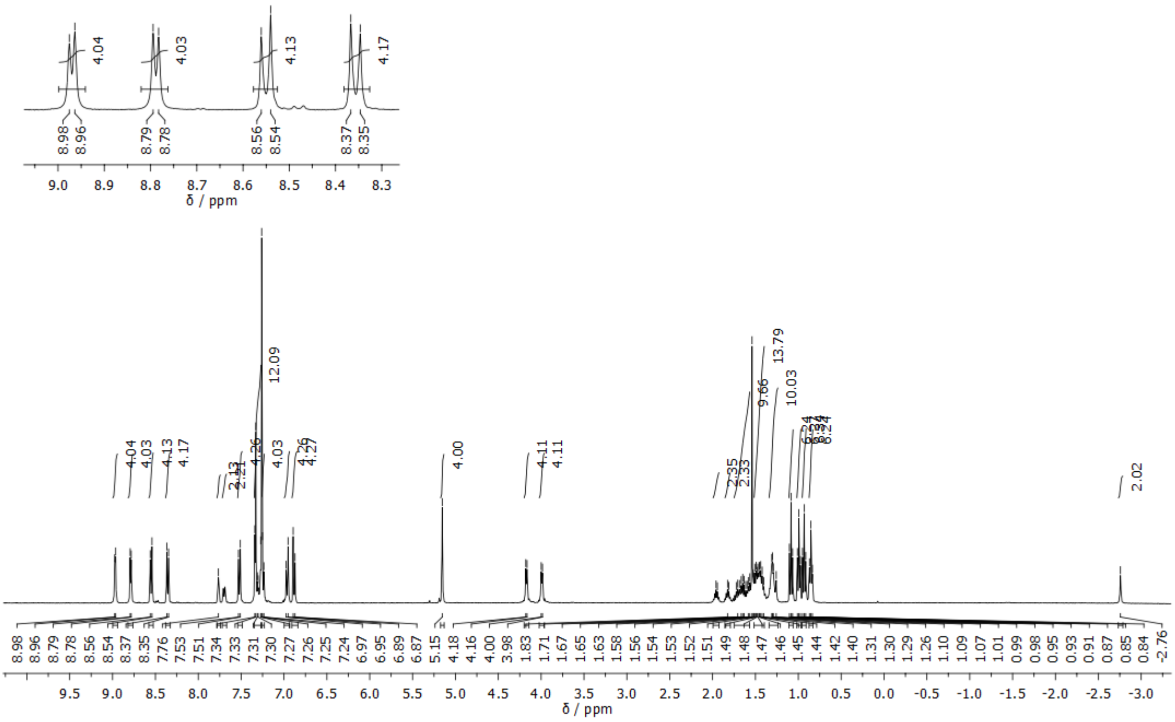

Figure 17.^{1}H-NMR, 400 MHz, $CDCl_3$.

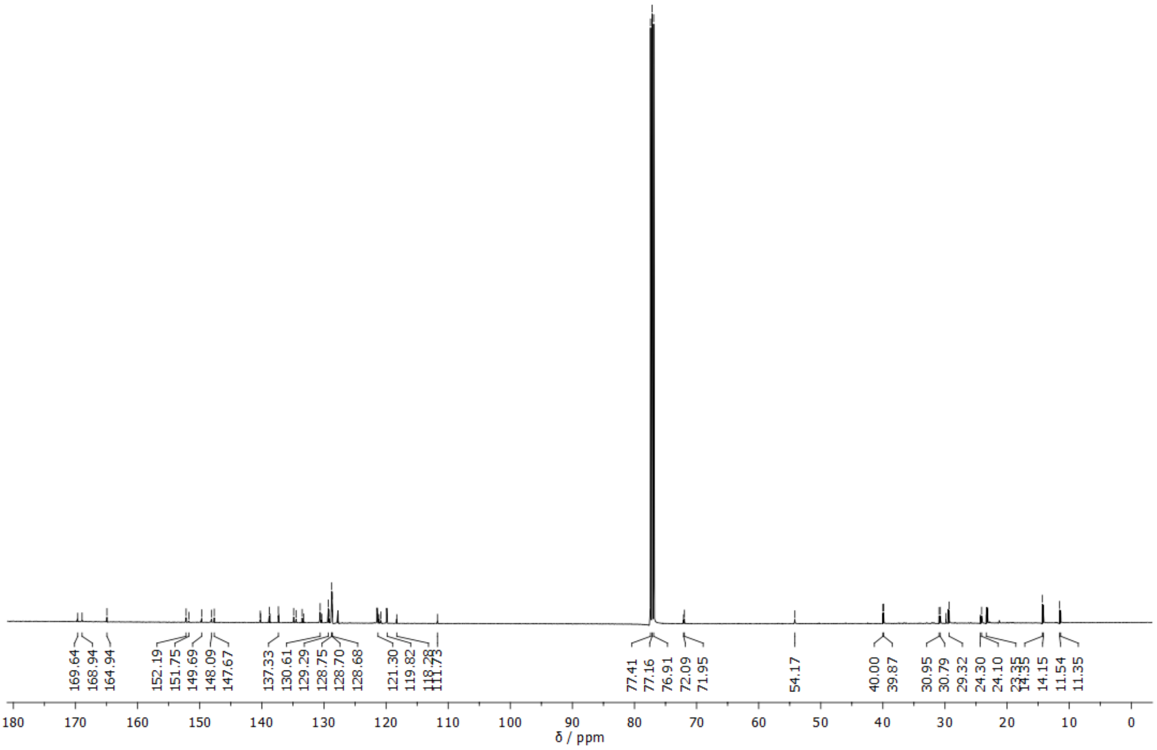

Figure 18.^{13}C-NMR, 100 MHz, $CDCl_3$.

Characterization of 11

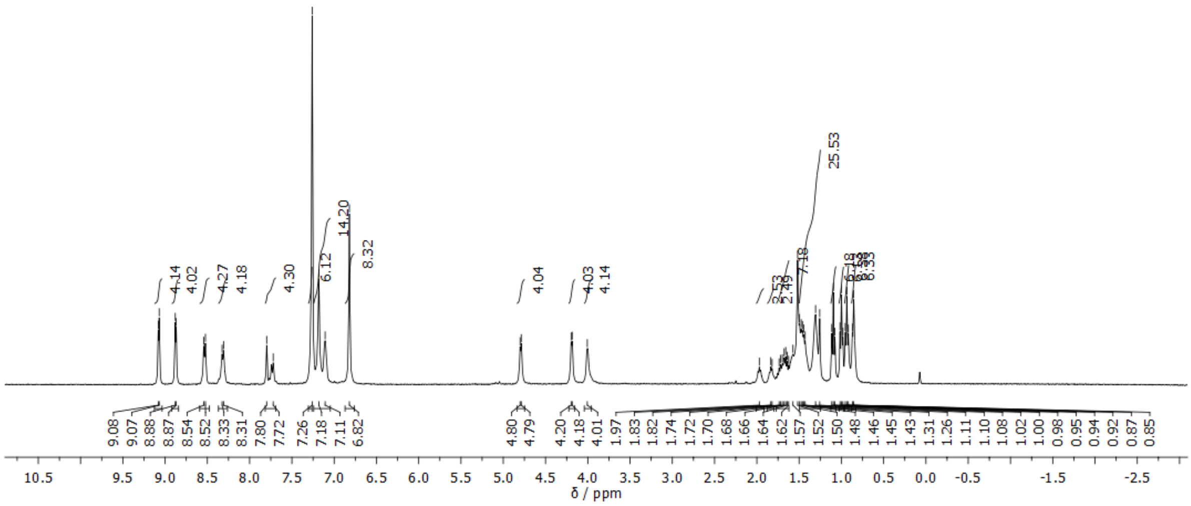

Figure 19.^{1}H-NMR, 400 MHz, CDCl3.

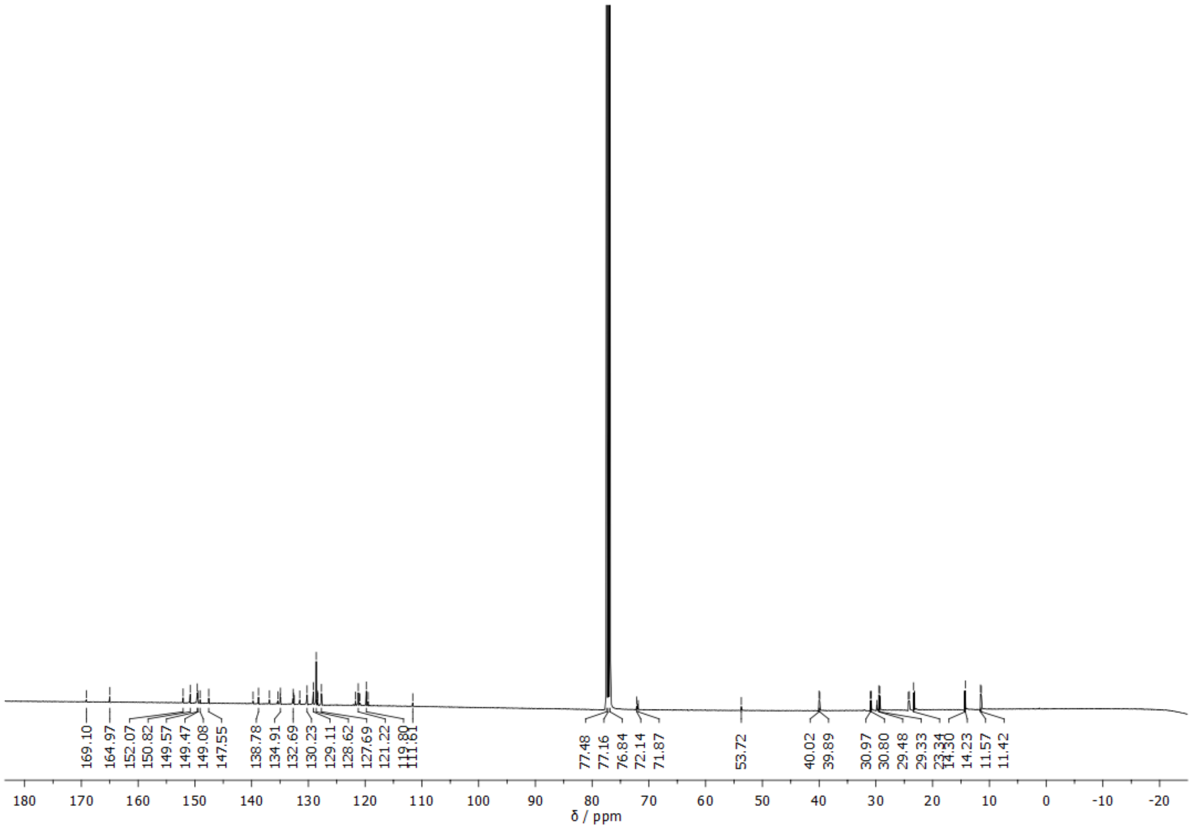

Figure 20.^{13}C-NMR, 100 MHz, CDCl3.

Characterization of 12-H_2

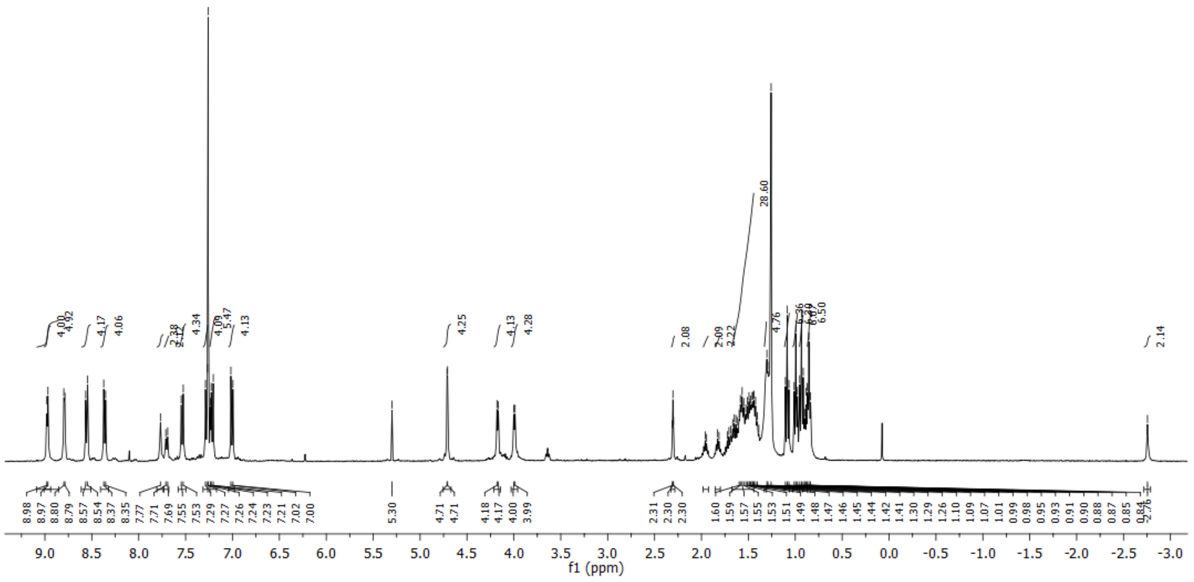

Figure 21.^{1}H-NMR, 400 MHz, $CDCl_3$.

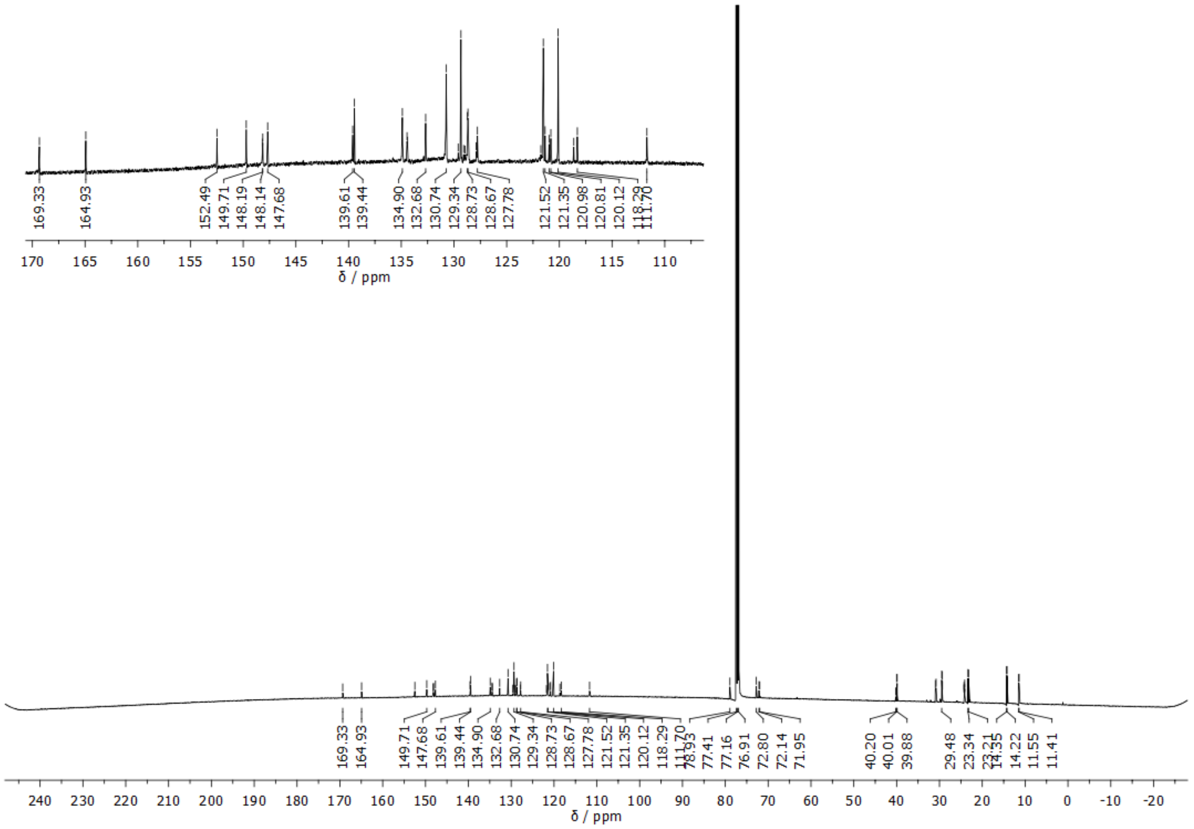

Figure 22.^{13}C-NMR, 100 MHz, $CDCl_3$.

Characterization of 12

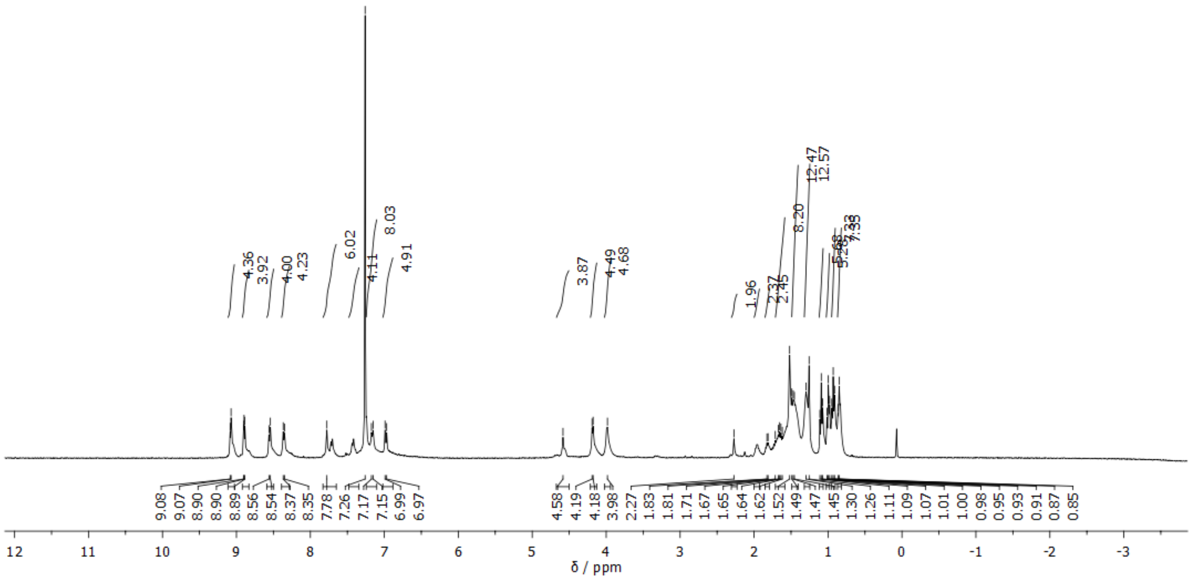

Figure 23.^{1}H-NMR, 400 MHz, $CDCl_3$.

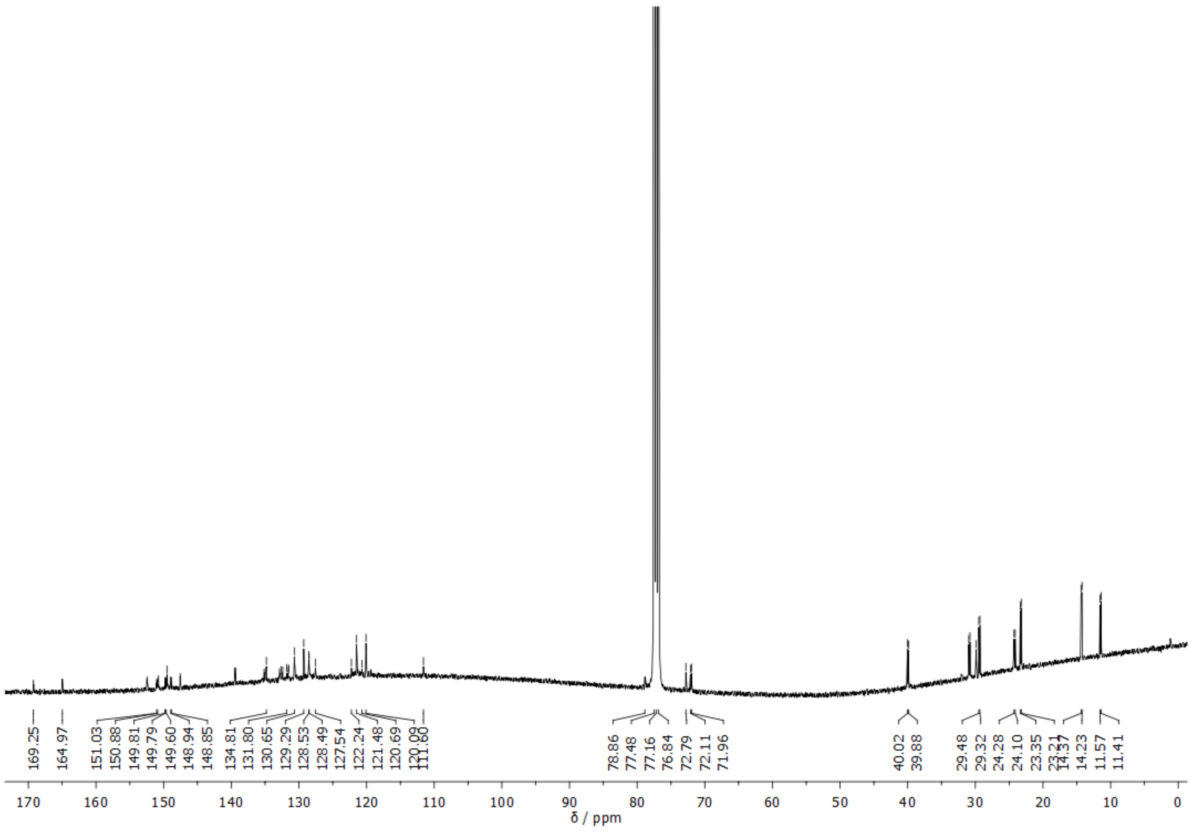

Figure 24.^{13}C-NMR, 100 MHz, $CDCl_3$.

Characterization of 13·DABCO

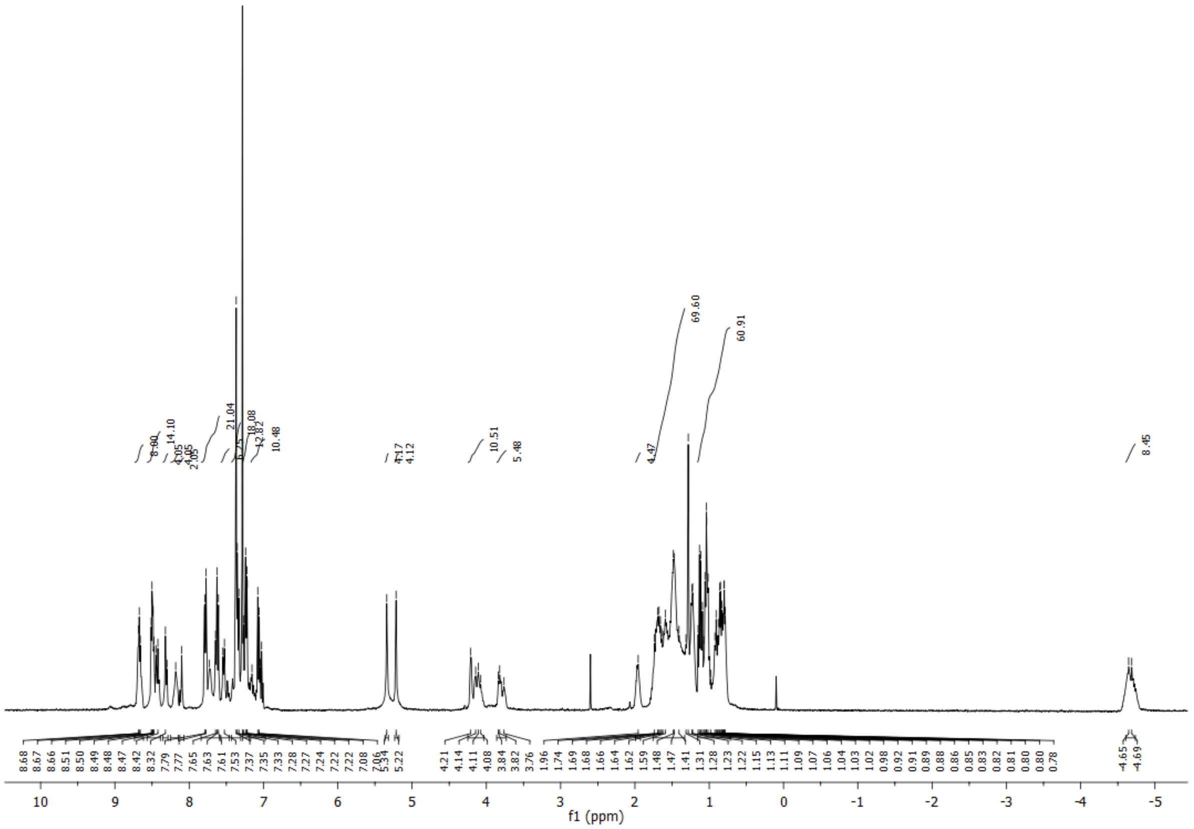

Figure 25.^{1}H-NMR, 400 MHz, $CDCl_3$.

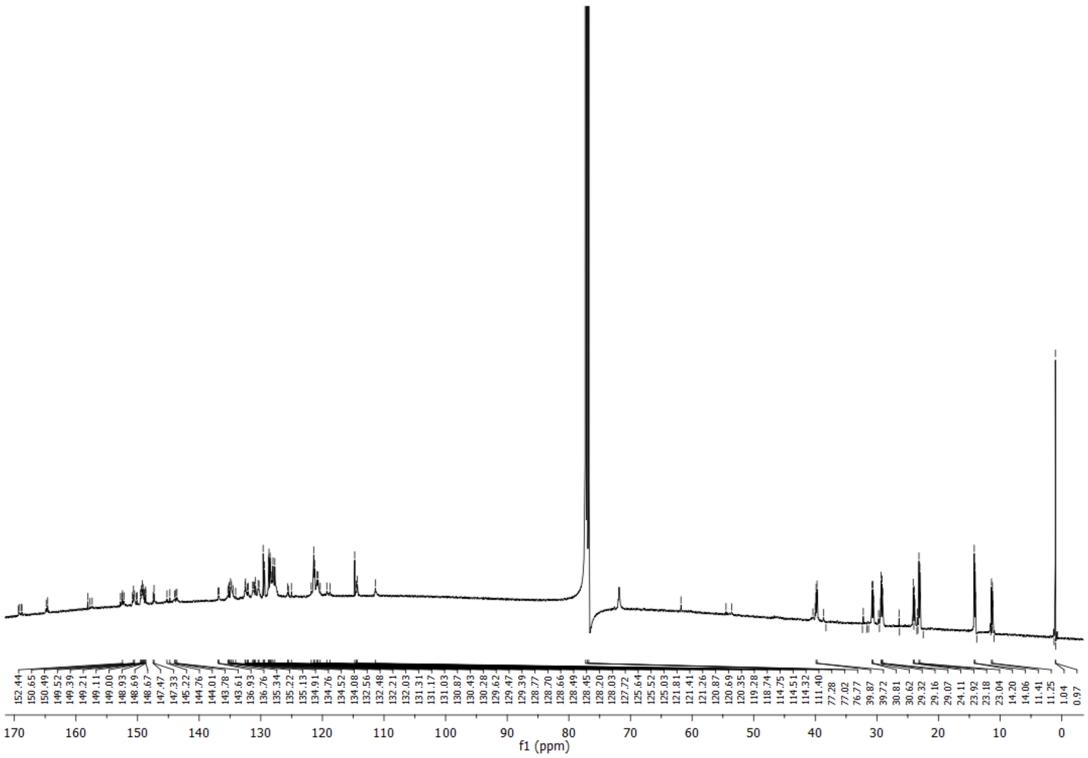

Figure 26.^{13}C-NMR, 100 MHz, $CDCl_3$.

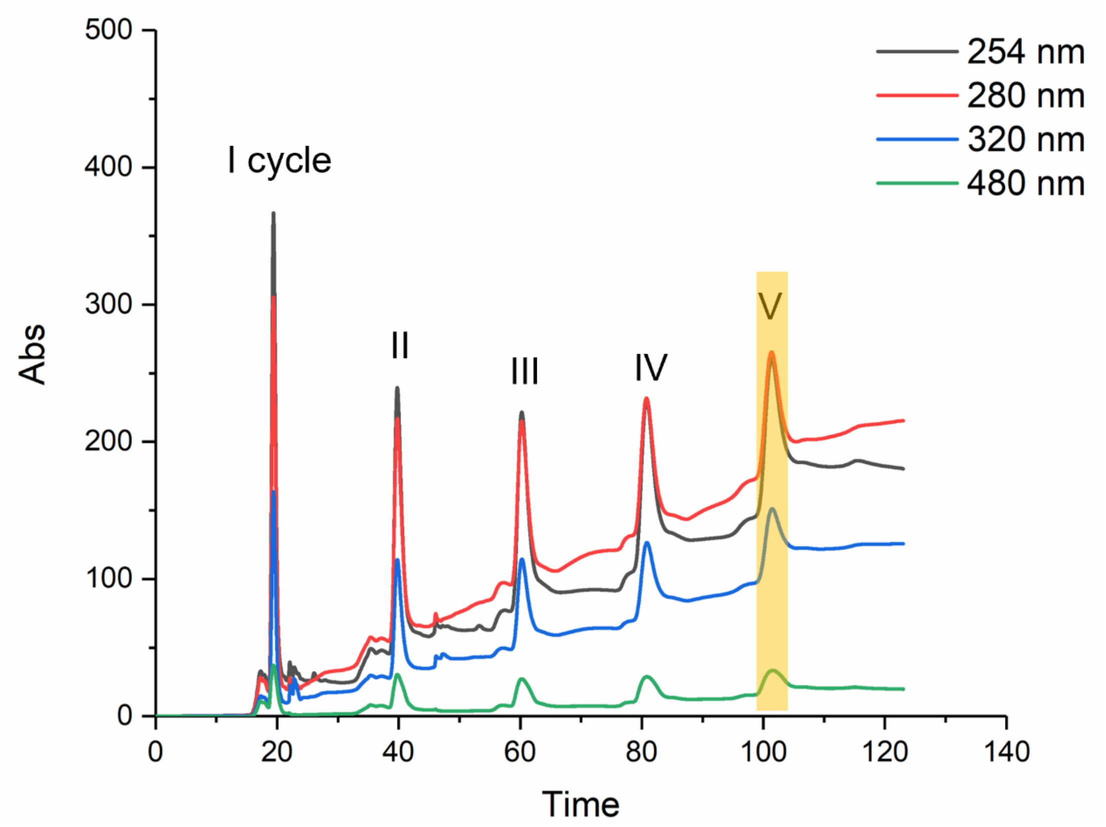

Figure 27. Gel permeation chromatography of **13·DABCO** in $CHCl_3$. The purification has been carried out in recycling mode and **13·DABCO** was isolate after five cycles.

Characterization of *p*15·DABCO - *a*15·DABCO

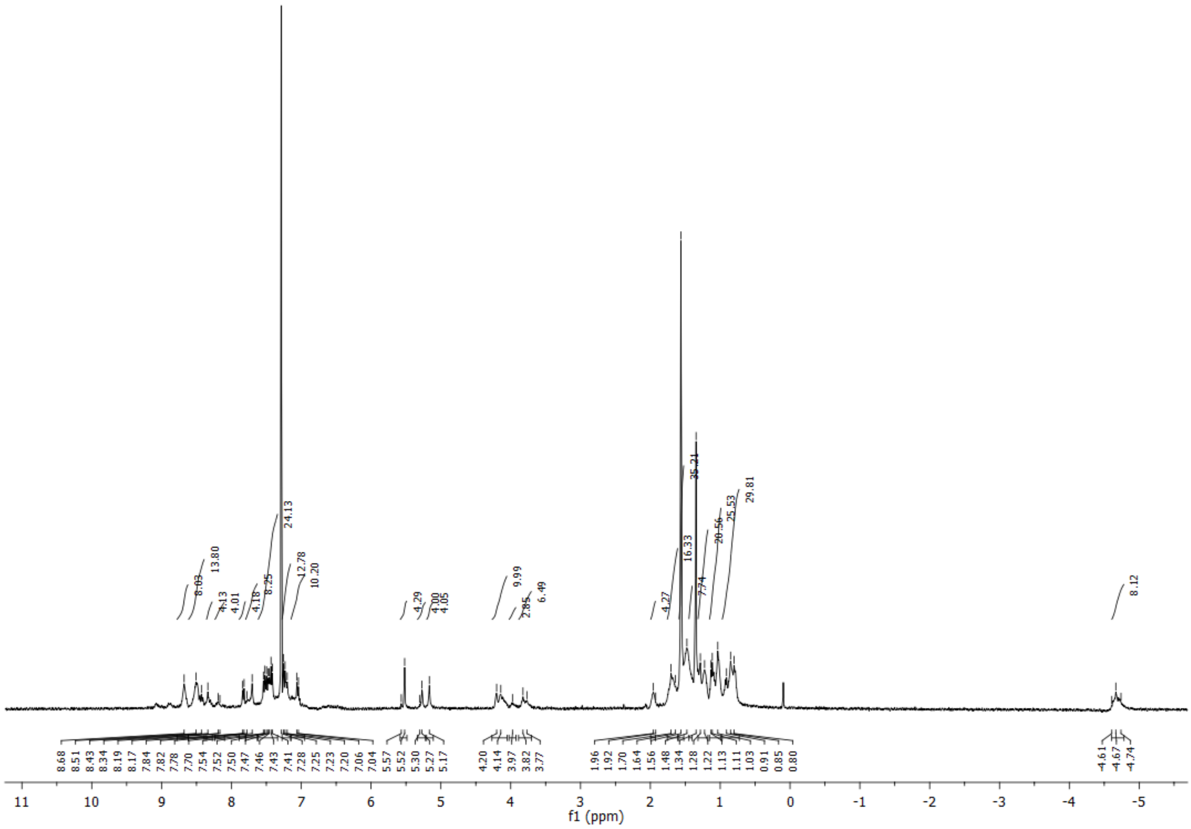

Figure 28. ^{1}H-NMR, 400 MHz, $CDCl_3$.

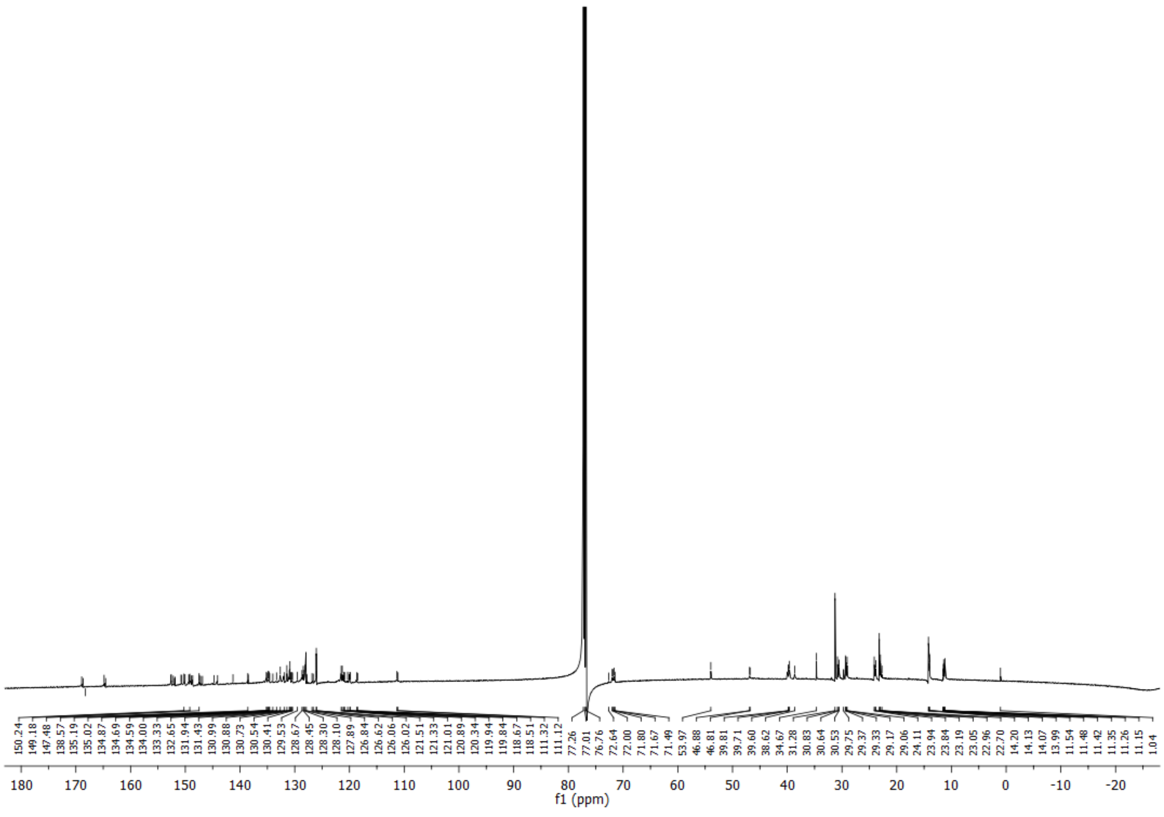

Figure 29. ^{13}C-NMR, 100 MHz, $CDCl_3$.
